# Supplementary material for: Transcriptomic meta-signatures identified in Anopheles gambiae populations reveal previously undetected insecticide resistance mechanisms
Source: Nat Commun. 2018 Dec 11;9:5282. doi: 10.1038/s41467-018-07615-x (PMC6290077; doi:10.1038/s41467-018-07615-x)
Supplement: Supplementary file 1 — Description of Additional Supplementary Files [file 41467_2018_7615_MOESM1_ESM.pdf]

Description of Additional Supplementary Files

File Name: Supplementary Data 1
Description: Significant Transcripts after dsMet Knockdown. VectorBase ID, Gene Description, Log2 Fold Change, Adjusted p-value and Fold Change for each significant (p<=0.05) transcript from microarray experiment comparing dsMet and dsGFP control adult female Tiassale mosquitoes.
