# Supplementary material for: Transcriptomic meta-signatures identified in Anopheles gambiae populations reveal previously undetected insecticide resistance mechanisms
Source: Nat Commun. 2018 Dec 11;9:5282. doi: 10.1038/s41467-018-07615-x (PMC6290077; doi:10.1038/s41467-018-07615-x)
Supplement: Supplementary file 4 — Supplementary Information [file 41467_2018_7615_MOESM4_ESM.pdf]

Transcriptomic meta-signatures identified in *Anopheles gambiae* populations reveal previously undetected insecticide resistance mechanisms.

Ingham et al.

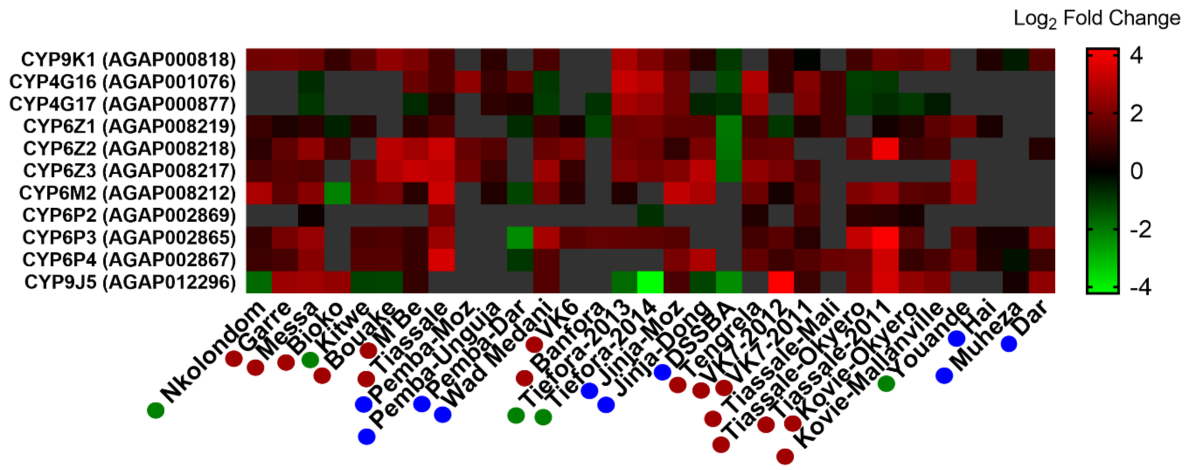

Supplementary Figure 1: Heatmap showing the $\log_2$ fold change for each cytochrome p450 previously reported to be involved in insecticide resistance. $\log_2$ fold change for 11 cytochrome p450s previously linked to insecticide resistance in each or the 31 microarray experiments used in IR-TEx. *CYP6M2*, *CYP6P2*, *CYP6P3, CYP6P4, CYP6Z1, CYP6Z2, CYP6Z3* and *CYP9J5* have all been shown to bind directly to or metabolise at least one class of insecticides [20,22,30,68,69]. *CYP4G16* catalyses the final step of hydrocarbon biosynthesis and has been shown to be strongly associated with pyrethroid resistance, alongside its paralog *CYP4G17* [51]. *CYP9K1* has been linked to a selective sweep in Mali associated with insecticide usage [70]. Red circles represent *An. coluzzii*, green *An. gambiae* and blue *An. arabiensis.* Grey shading demonstrates non-significance. It should be noted that some co-hybridisation of *CYP6Z2* and *CYP6Z3* is possible due to high probe similarity (Supplementary Table 4).

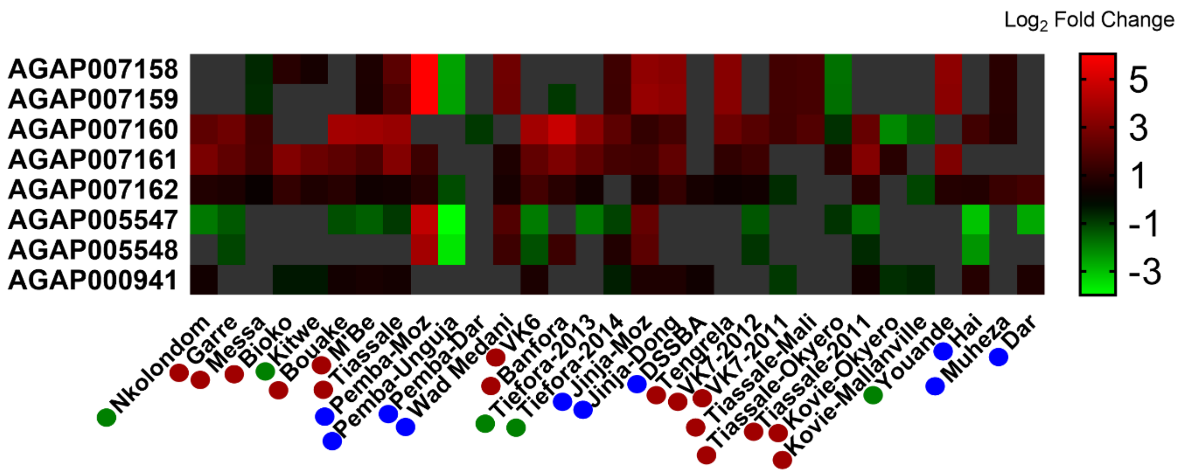

Supplementary Figure 2: Heatmap showing the $\log_2$ fold change for each α-crystallin transcript in the 2L cluster.

Log_2 fold change for each of the five α-crystallins clustered on 2L in the *An. gambiae* genome for each of the 31 datasets used in the IR-TEx app. Red circles represent *An. coluzzii*, green *An. gambiae* and blue *An. arabiensis.* Grey shading demonstrates non-significance.

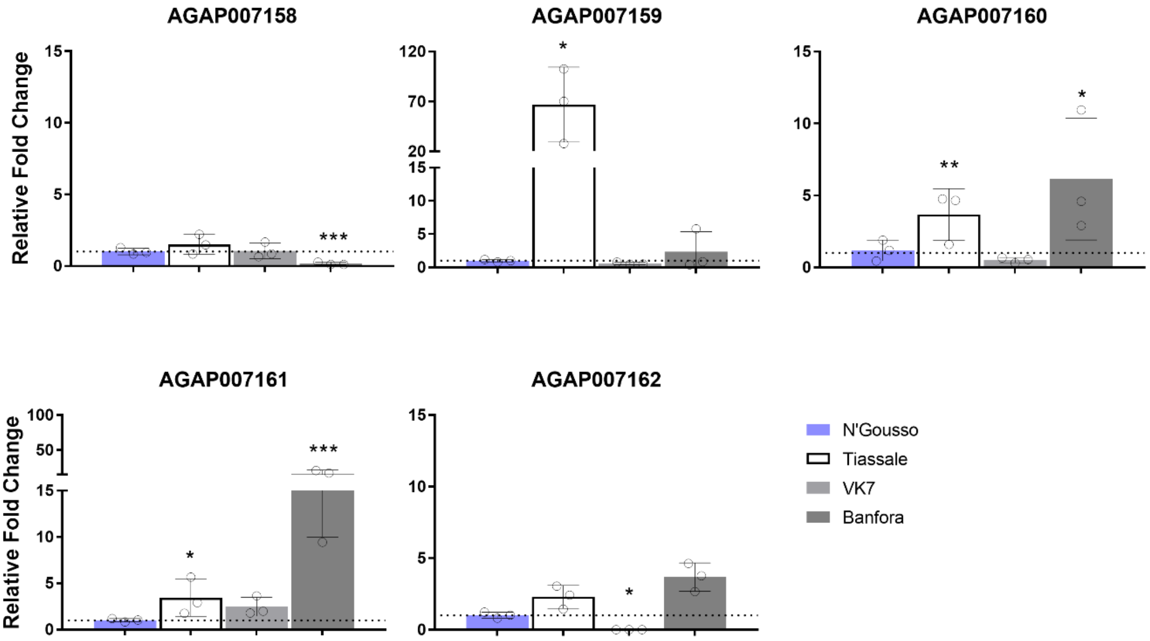

Supplementary Figure 3: qPCR analysis of 5 of the α-crystallins clustered on 2L from multiple resistant populations. qPCR results of three 3-5 day old, unexposed, pyrethroid resistant *Anopheles* populations compared to the lab susceptible N'Gousso, three biological replicates and three technical replicates were used for each gene. Relative fold change (y), and resistant populations (x). Standard deviation bars are shown, with significance * $p \leq 0.05$, ** $p \leq 0.01$, *** $p \leq 0.001$ as calculated by an ANOVA with Dunnett's *post hoc* test and in the cases of AGAP007162 and AGAP007159 a Kruskall-Wallis with Dunn's *post hoc* test.

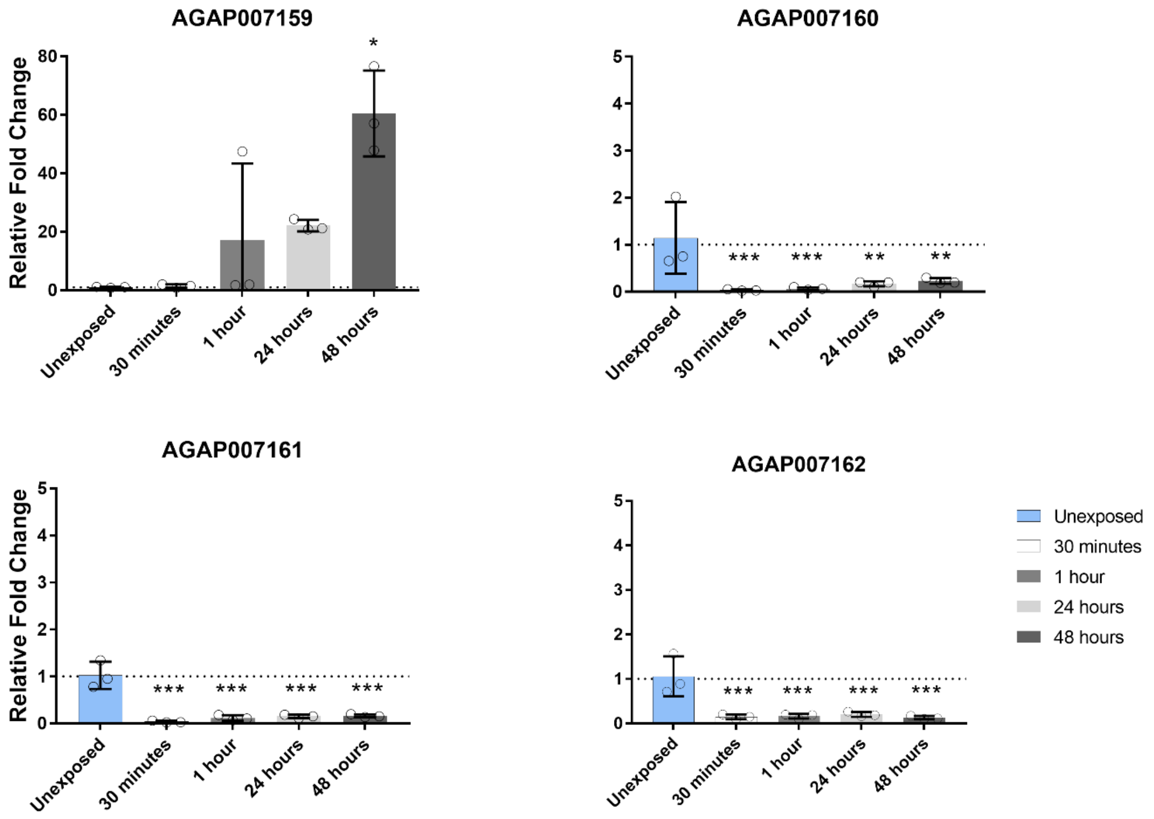

Supplementary Figure 4: Induction of α-crystallin family after exposure to deltamethrin. The 4 α-crystallins showing differential expression in at least one resistant population were screened in Tiassale mosquitoes at four time points following deltamethrin exposure. qPCR was performed on 3-5 day old females exposed to WHO 0.05% deltamethrin papers for 1 hours, at 30 minutes, 1 hour, 24 hours and 48 hours post-exposure. All data is normalised relative to 3-5 day old unexposed Tiassalé female mosquitoes, three biological replicates and three technical replicates were used for each. Relative fold change (y-axis) and time points (x-axis) are shown. Standard deviation bars are shown, with significance * $p \leq 0.05$, ** $p \leq 0.01$, *** $p \leq 0.001$ as calculated by ANOVA with Dunnett's *post hoc* test, except AGAP007159 which was calculated by Kruskall-Wallis with Dunn's *post hoc* test.

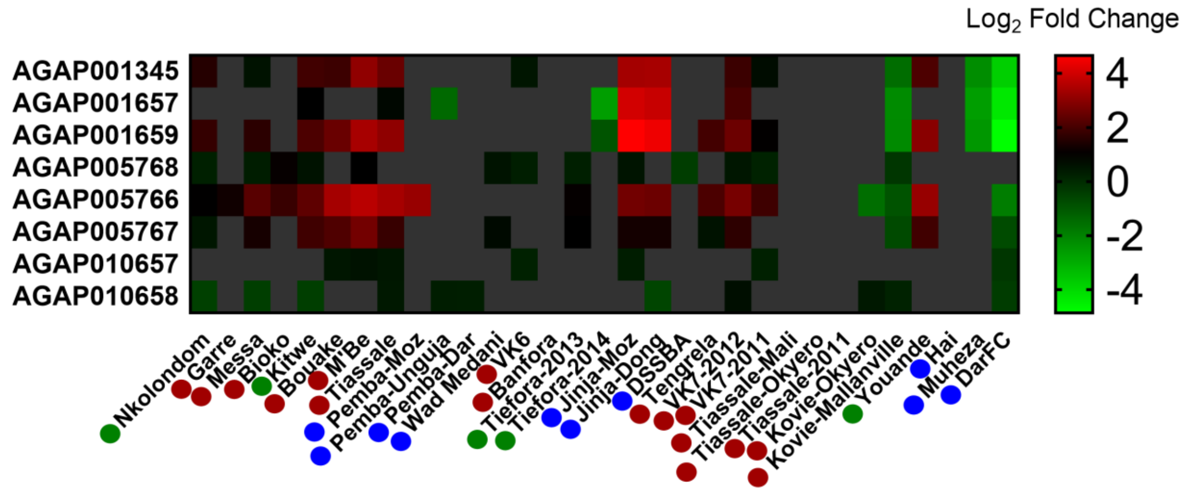

Supplementary Figure 5: Heatmap showing the log$_2$ fold change for each hexamerin transcript. Log$_2$ fold change for each hexamerin represented in the *An. gambiae* genome for each of the 31 datasets used in the IR-TEx app. Red circles represent *An. coluzzii*, green *An. gambiae* and blue *An. arabiensis.* Grey shading demonstrates non-significance.

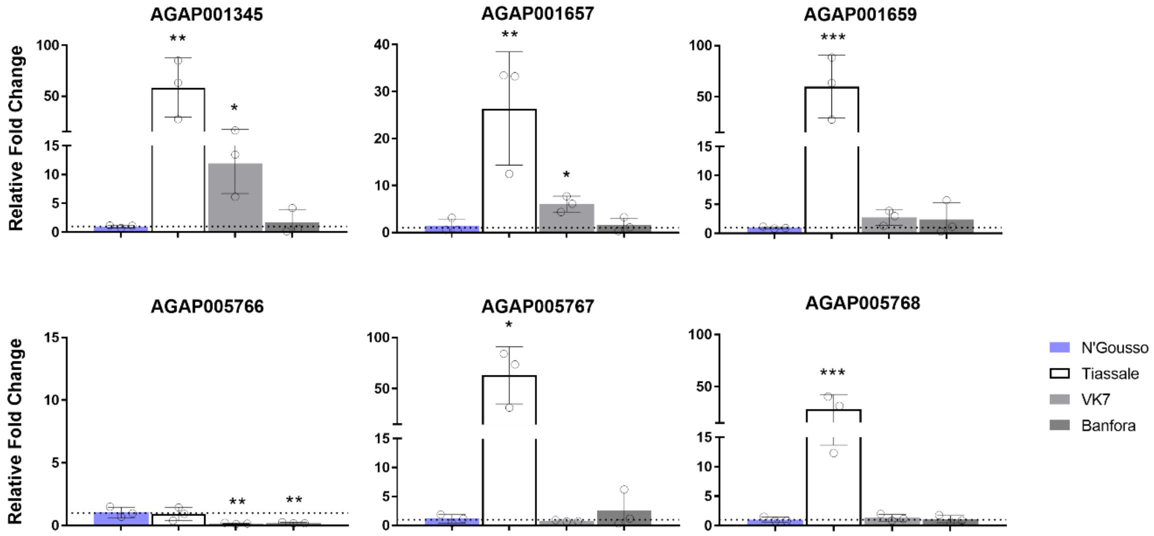

Supplementary Figure 6: qPCR analysis of hexamerin family from multiple resistant populations. qPCR results of three 3-5 day old, unexposed, pyrethroid resistant *Anopheles* populations compared to the lab susceptible N'Gousso, three biological replicates and three technical replicates were used for each gene. Relative fold change (y), and resistant populations (x). Standard deviation bars are shown, with significance * $p \leq 0.05$, ** $p \leq 0.01$, *** $p \leq 0.001$ as calculated by ANOVA with Dunnett's *post hoc* test.

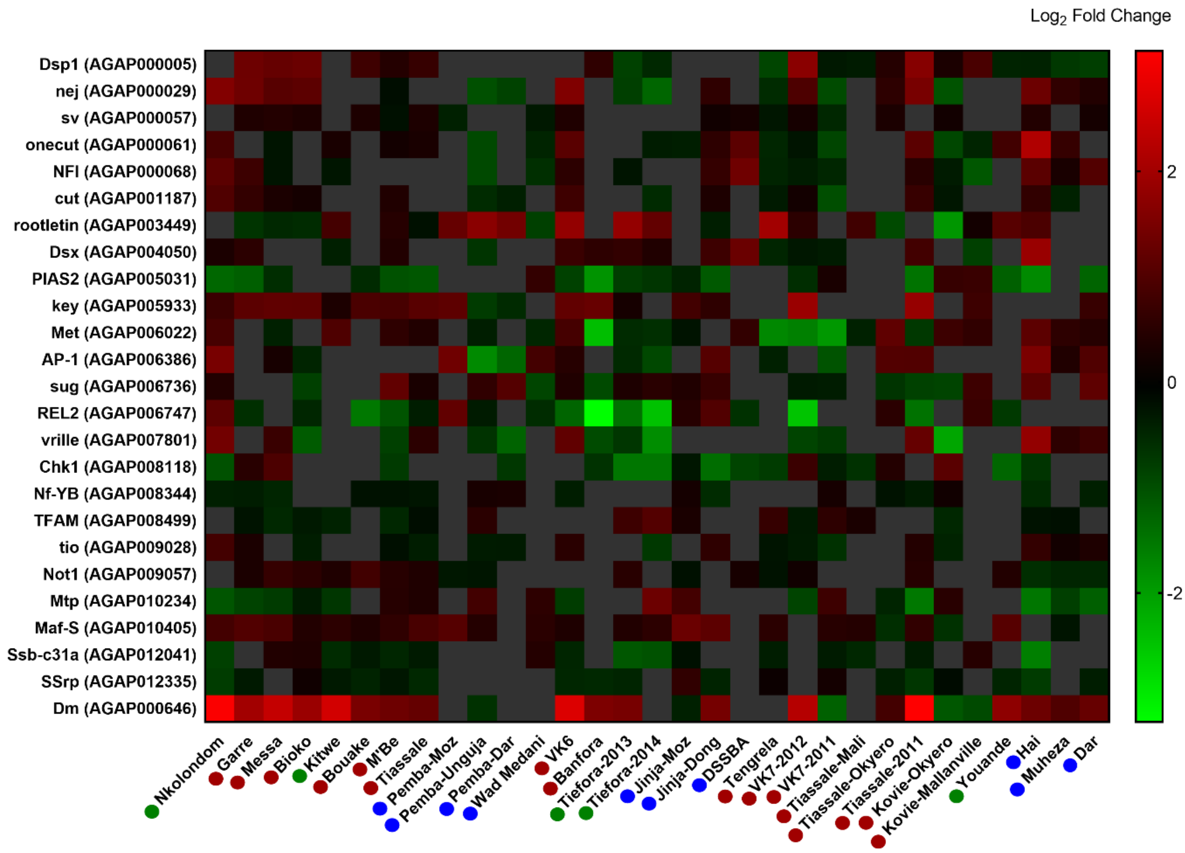

Supplementary Figure 7: Heatmap showing the $\log_2$ fold change for each transcription factor significantly differential in at least 50% of the experiments.

Log_2 fold change for each hexamerin represented in the *An. gambiae* genome for each of the 31 datasets used in the IR-TEx app. Red circles represent *An. coluzzii*, green *An. gambiae* and blue *An. arabiensis.* Grey shading demonstrates non-significance.

Supplementary Table 1: Meta-data associated with each of the 31 analysed microarray experiments

Name in IR-Tex	Nameof Resistant Population	Species	Country of origin of resistant population	Resistant Collection Location	Co-ordiantes of Sample Site	Year resistant mosquitoes collectd	Insectide mosquitoes were exposed to prior to RNA extraction from survivors	WHO Paper Exposure Time pre-RNA extraction	Resistant population mortality	Description of mosquito breeding site	Frequency of kdr alleles in population	Susceptible Population	Country of origin of susceptible population	Reference	ArrayExpress Accession Number
GarreFC	Garre	coluzzii	Cameroon	Garre, Youande	3.85,11.5*	2009	DDT	1 hour	38%	Polluted	78% 1014F*	N'Gousso*	Cameroon	7	E-MTAB-1382
MessaFC	Messa	coluzzii	Cameroon	Messa, Youande	3.85,11.5*	2009	DDT	1 hour	54.30%	Unpolluted	29% 1014F*	N'Gousso*	Cameroon	7	E-MTAB-1382
NkolondomFC	Nkolondom	gambiae	Cameroon	Nkolondom, Youande	3.85,11.5*	2009	DDT	1 hour	3.90%	Urban farming	1014F*	Kisumu*	Kenya	7	E-MTAB-1382
BiokoFC	Bioko	coluzzii	Equatorial Guinea	Malabo	3.75,8.73	2011	Deltamethrin	1 hour	40%		58% 1014F^	N'Gousso*	Cameroon	8	VectorBase
KitweFC	Kitwe	gambiae	Zambia	Kitwe, Copperbelt Province	-12.82,28.22	2011-2012	Deltamethrin	1 hour	38.40%		91% 1014F^	Kisumu*	Kenya	9	See Paper
BouakeFC	Bouake	coluzzii	Cote D'Ivoire	Bouake	7.69,-5.04	2010	Deltamethrin	1 hour	18%		100% 1014F"	N'Gousso*	Cameroon	10	E-MTAB-3212
TiassaleFC	Tiassale	coluzzii	Cote D'Ivoire	Tiassale	5.90,-4.83	2010	Deltamethrin	1 hour	15%		82% 1014F"	N'Gousso*	Cameroon	10	E-MTAB-3210
MBeFC	MBe	coluzzii	Cote D'Ivoire	M'Be	-5.29,7.41	2010	Deltamethrin	1 hour	26%		Unknown	N'Gousso*	Cameroon	10	E-MTAB-3211
PembaDarFC	Pemba	arabiensis	Tanzania	Pemba, Zanzibar	-5.03,39.78	2012	Lambda-Cyhalothrin	1 hour	48.90%	Rural inland	Unknown	Dar*	Tanzania	11	E-MTAB-2074
PembaUngFC	Pemba	arabiensis	Tanzania	Pemba, Zanzibar	-5.03,39.78	2012	Unexposed		48.9% (Lambda)	Rural inland	Unknown	Unguja	Tanzania	11	E-MTAB-2074
PembaMozFC	Pemba	arabiensis	Tanzania	Pemba, Zanzibar	-5.03,39.78	2011	Lambda-Cyhalothrin	1 hour	34%	Rural inland	Unknown	Moz*	Mozambique	11	E-MTAB-2075
WadMedaniFC	Wad Medani	arabiensis	Sudan	Wad Medani	14.39,33.54	2010	Permethrin	1 hour	60-80%	Subsistence agriculture	81% 1014F*	Dong*	Sudan	12	E-MEXP-3656
VK6FC	VK6	coluzzii	Burkina Faso	Vallee de Kou	11.39,-4.41*	2010	Unexposed		20% (delta)	Wooded savannah	73.1% 1014F*	N'Gousso*	Cameroon	13	E-MTAB-1083
BanforaFC	Banfora	coluzzii	Burkina Faso	Banfora	10.64,-4.76	2014	Unexposed		0% (perm)		Unknown	N'Gousso*	Cameroon	Unpublished data	E-MTAB-6498
Tiefora13FC	Tiefora	gambiae	Burkina Faso	Banfora	10.64,-4.76	2013	Unexposed		33% (perm)		95% 1014F"	Kisumu*	Kenya	Unpublished data	E-MTAB-6499
Tiefora14FC	Tiefora	gambiae	Burkina Faso	Banfora	10.64,-4.76	2014	Unexposed		0% (perm)		Unknown	Kisumu*	Kenya	Unpublished data	E-MTAB-6500
JinjaMozFC	Jinja	arabiensis	Uganda	Jinja district-Walukuba	1.03,34.24*	2011	Permethrin	1 hour	56.90%	Urban centre	0.07% 1014S^	Moz*	Mozambique	14	E-MTAB-1873
JinjaDongFC	Jinja	arabiensis	Uganda	Jinja district-Walukuba	1.03,34.24*	2011	Permethrin	1 hour	56.90%	Urban centre	0.07% 1014S^	Dong*	Sudan	14	E-MTAB-1873
DSSBAFC	DSSBA	arabiensis	Burkina Faso	Dioulassoba	11.18,4.29*	2009	DDT	1 hour	65.8-70.4%	Polluted, urban district	43% 1014S*	Moz*	Mozambique	15	E-MTAB-1224
VK7_11FC	VK7	coluzzii	Burkina Faso	Vallee de Kou	11.65,-4.68*	2011	Deltamethrin	4 hour	0%	Wooded savannah	82.3% 1014F^	Mali*	Mali	10	E-MTAB-2859
VK7_12FC	VK7	coluzzii	Burkina Faso	Vallee de Kou	11.65,-4.68*	2012	Deltamethrin	10 hour	0%	Wooded savannah	88% 1014F^	N'Gousso*	Cameroon	10	E-MTAB-2875
TengrelaFC	Tengrela	coluzzii	Burkina Faso	Tengrela	10.67,-4.83*	2012	Unexposed		50%		87.7% 1014F^	Mali*	Mali	10	E-MTAB-2875
TiassaleMaliFC	Tiassale	coluzzii	Cote D'Ivoire	Tiassale	5.90,-4.83	2011	Unexposed		23% (perm)		83% 1014F^	Mali*	Mali	16	E-MTAB-1903
TiassaleOkyeroFC	Tiassale	coluzzii	Cote D'Ivoire	Tiassale	5.90,-4.83	2011	Unexposed		23% (perm)		83% 1014F^	Okyero	Ghana	16	E-MTAB-1903
Tiassale11FC	Tiassale	coluzzii	Cote D'Ivoire	Tiassale	5.90,-4.83	2011	Unexposed		23% (perm)		83% 1014F^	N'Gousso*	Cameroon	16	E-MTAB-1903
KovieMalFC	Kovie	coluzzii	Togo	Kovie	6.34,1.11	2011	Unexposed		Unknown		Unknown	Malanville	Benin	16	E-MTAB-1889
KovieOkyFC	Kovie	coluzzii	Togo	Kovie	6.34,1.11	2011	Unexposed		Unknown		Unknown	Okyero	Ghana	16	E-MTAB-1889
YouandeFC	Youande	gambiae	Cameroon	Youande	3.85,11.5*	2010-2013	Bendiocarb	1 hour	53.2-100%	Cultivated sites	Unknown	Kisumu*	Kenya	17	E-MTAB-1563
DarFC	Dar	arabiensis	Tanzania	Dar Es Salaam	39.78,-6.81	2011	Unexposed		100% (Delta)	Urban	Unknown	Ifakara*	Tanzania	18	E-MEXP-3987
MuhezaFC	Muheza	arabiensis	Tanzania	Muheza	39.07,-6.82	2011	Unexposed		100% (Delta)	Non-Polluted	Unknown	Ifakara*	Tanzania	18	E-MEXP-3987
HaiFC	Hai	arabiensis	Tanzania	Hai District	37.23,-3.31	2011	Unexposed		84-87% (Delta)	Agriculture	Unknown	Ifakara*	Tanzania	18	E-MEXP-3987
					Lattitute, Longitude. * represents exact collection site as reported by the relevant publication. Others are estimations based on town/city of collection				If unexposed mosquitoes used in microarray experiment, then the insecticide used for motality calculations in the study are shown in parentheses, otherwise mortality data corresponds to insecticide used in exposure.		* kdr frequency in survivours. ^ kdr frequency in collected mosquitoes. " Unpublished. Percentages are given where recorded in the study. No percentage indicates presence of mutation but no frequency reported. Unknown indicate no data given on the population	* Indicates a fully susceptible population with pyrethroid mortality > 90%. Unguja was collected from Unguja Island at the same time as the Pemba samples, these exhibited suspected resistance (81.9%-88.4%) to permethrin. Okyero and Malanville are carbamate susceptible populations and are assumed susceptible to pyrethroids			

Supplementary Table 2: Transcripts showing consistent fold change directionality across all pyrethroid exposed arrays. VectorBase ID, Transcript name or description, average fold changes across all the arrays and fold changes of all relevant data sets are displayed. Transcripts consistently up-regulated trend are highlighted in red, and down-regulated in green.

VectorBase ID	Transcript Type	Average Fold Change	BiokoFC	KitweFC	BouakeFC	MBeFC	TiassaleFC	PembaMozFC	PembaDarFC	WadMedaniFC	JinjaMozFC	JinjaDongFC	VK72012FC	VK72011FC
AGAP001659-RA	hexamerin [Source:VB Community Annotation]	7.630	2.181	4.213	5.634	10.411	8.192	3.427	1.922	1.417	25.032	21.271	5.826	2.038
AGAP008217-RA	CYP6Z3	5.909	1.659	4.935	10.037	12.805	9.902	3.797	1.279	6.352	4.874	8.548	4.093	2.629
AGAP008217-RA	CYP6Z3	5.672	1.659	4.935	10.037	12.805	9.902	3.797	1.279	6.352	4.874	8.548	4.093	2.629
AGAP013061-RA	Haemolymph juvenile hormone binding	4.992	9.368	2.477	7.311	12.476	13.812	1.942	1.734	2.527	1.068	1.458	4.336	1.393
AGAP008217-RA	CYP6Z3	4.629	1.659	4.935	10.037	12.805	9.902	3.797	1.279	6.352	4.874	8.548	4.093	2.629
AGAP001345-RA	hexamerin [Source:VB Community Annotation]	4.503	1.473	3.785	3.611	8.285	5.644	3.050	1.348	1.184	10.075	10.393	3.557	1.635
AGAP002603-RA	elongation factor 1 alpha-like protein [Source:VB Community Annotation]	4.020	17.965	1.334	4.399	1.986	7.581	1.063	1.004	1.003	1.034	1.013	4.995	4.860
AGAP007161-RA	alpha-crystallin chain A [Source:VB Community Annotation]	3.938	8.439	5.670	4.479	3.341	8.625	2.661	1.020	1.618	2.861	4.787	2.688	1.071
AGAP008217-RA	CYP6Z3	3.881	1.659	4.935	10.037	12.805	9.902	3.797	1.279	6.352	4.874	8.548	4.093	2.629
AGAP008438-RA		3.442	2.136	3.625	3.803	4.237	2.248	1.229	1.483	6.682	2.973	5.462	4.877	2.546
AGAP001706-RA		3.180	1.644	2.699	1.319	1.656	2.521	6.042	1.441	1.710	2.812	6.345	1.792	8.183
AGAP008021-RA	CYP12F2	3.152	1.276	10.790	1.912	1.774	1.753	1.340	1.137	2.234	3.724	8.768	1.458	1.405
AGAP008021-RA	CYP12F2	3.131	1.276	10.790	1.912	1.774	1.753	1.340	1.137	2.234	3.724	8.768	1.458	1.405
AGAP009766-RA		3.016	2.223	1.027	2.141	2.955	1.427	6.395	1.619	5.160	1.347	4.893	3.911	3.089
AGAP008021-RA	CYP12F2	2.901	1.276	10.790	1.912	1.774	1.753	1.340	1.137	2.234	3.724	8.768	1.458	1.405
AGAP008021-RA	CYP12F2	2.852	1.276	10.790	1.912	1.774	1.753	1.340	1.137	2.234	3.724	8.768	1.458	1.405
AGAP004164-RC	GSTD1_6	2.706	1.818	1.832	2.611	4.220	2.417	1.099	1.342	1.445	4.807	6.358	2.598	1.927
AGAP006985-RA		2.548	2.008	3.714	1.634	1.152	3.883	1.395	1.813	1.753	4.259	5.270	1.259	2.434
AGAP004382-RA	GSTD3	2.352	1.208	1.849	1.660	1.287	2.255	2.369	1.654	2.195	4.929	3.762	2.304	2.756
AGAP004382-RA	GSTD3	2.347	1.208	1.849	1.660	1.287	2.255	2.369	1.654	2.195	4.929	3.762	2.304	2.756
AGAP004382-RA	GSTD3	2.317	1.208	1.849	1.660	1.287	2.255	2.369	1.654	2.195	4.929	3.762	2.304	2.756
AGAP011698-RA	deoxyribonuclease I [Source:VB Community Annotation]	2.315	3.522	2.418	1.883	5.002	2.365	1.540	1.015	1.455	1.125	1.104	3.487	2.857
AGAP002940-RA	coiled-coil-helix-coiled-coil-helix domain containing 4 [Source:VB Community Annotation]	2.253	3.939	1.215	2.658	2.495	2.964	1.011	1.113	1.177	1.428	3.402	2.428	3.203
AGAP004382-RA	GSTD3	2.237	1.208	1.849	1.660	1.287	2.255	2.369	1.654	2.195	4.929	3.762	2.304	2.756
AGAP004164-RC	GSTD1_6	2.229	1.818	1.832	2.611	4.220	2.417	1.099	1.342	1.445	4.807	6.358	2.598	1.927
AGAP009743-RA	ribonuclease Z [Source:VB Community Annotation]	1.944	1.157	1.879	2.416	3.821	1.906	1.441	1.238	2.080	2.025	2.606	1.374	1.381
AGAP009946-RA	GSTMS3	1.931	1.667	2.758	1.979	1.271	1.509	1.517	1.428	1.121	2.011	2.905	3.007	1.995
AGAP010932-RA		1.893	3.445	1.617	2.056	2.551	1.662	1.187	1.006	1.420	1.060	1.231	3.436	2.043
AGAP004113-RA	39S ribosomal protein L55, mitochondrial [Source:VB Community Annotation]	1.693	3.493	1.274	1.857	2.405	1.637	1.216	1.427	1.100	1.331	1.155	2.151	1.267
AGAP008761-RA	translocon-associated protein subunit gamma [Source:VB Community Annotation]	1.671	2.666	1.184	1.453	1.188	1.968	1.966	1.289	1.190	2.036	1.774	1.712	1.622
AGAP001072-RA		1.659	1.870	3.128	1.524	1.645	1.268	1.052	1.330	1.262	1.007	2.060	1.717	2.038
AGAP011318-RA	Synaptogyrin [Source:VB Community Annotation]	1.629	3.201	1.371	1.203	1.518	1.064	1.863	1.228	1.301	1.281	1.180	2.961	1.373
AGAP013008-RA		1.623	2.052	1.056	1.277	1.430	1.370	3.569	1.269	1.020	1.695	1.270	1.744	1.724
AGAP006364-RC	ABC-B (AGAP006364)	1.620	1.702	1.700	2.101	3.320	2.005	1.102	1.042	1.447	1.017	1.477	1.214	1.312
AGAP005055-RA	ion-transport peptide CHH-like [Source:VB Community Annotation]	1.620	1.114	1.454	1.748	2.478	1.761	2.495	1.170	1.845	1.282	1.502	1.404	1.182
AGAP004163-RA	GSTD7	1.552	1.188	1.204	2.086	1.679	2.104	2.019	1.236	1.333	1.371	1.811	1.050	1.466
AGAP004163-RA	GSTD7	1.546	1.188	1.204	2.086	1.679	2.104	2.019	1.236	1.333	1.371	1.811	1.050	1.466
AGAP004163-RB	GSTD7	1.543	1.153	1.159	2.042	1.758	2.063	2.284	1.204	1.182	1.331	1.966	1.008	1.371
AGAP006364-RB	ABC-B (AGAP006364)	1.541	1.608	1.593	2.420	3.118	2.549	1.080	1.072	1.493	0.912	1.552	1.264	1.280
AGAP006651-RA	peroxisomal 3,2-trans-enoyl-CoA isomerase [Source:VB Community Annotation]	1.530	2.474	1.329	1.283	1.201	1.163	1.034	1.213	1.304	1.219	1.066	2.718	2.358
AGAP007121-RA	cytochrome b5 protein [Source:VB Community Annotation]	1.515	0.759	2.130	2.350	1.627	2.365	4.678	0.831	2.144	2.055	6.699	1.822	1.504
AGAP012514-RA	iron-regulated short-chain dehydrogenase/reductase [Source:VB Community Annotation]	1.485	1.396	1.593	1.541	1.012	1.462	1.651	1.331	1.003	1.801	1.593	1.763	1.671
AGAP009192-RA	GSTE5	1.482	1.184	1.614	0.830	1.509	1.496	0.828	1.338	1.374	1.652	2.812	2.304	2.266
AGAP007366-RB		1.475	2.429	1.060	1.727	1.488	1.392	1.015	1.274	1.063	1.224	1.374	2.199	1.452
AGAP004598-RA	Phosphoserine aminotransferase [Source:UniProtKB/TrEMBL;Acc:Q5TRW7]	1.471	1.428	1.436	1.738	2.122	2.061	1.042	1.582	1.197	1.384	1.191	1.034	1.434
AGAP007366-RA		1.465	2.478	1.000	1.706	1.434	1.448	1.103	1.211	1.048	1.149	1.235	2.275	1.487
AGAP005768-RA	hexamerin [Source:VB Community Annotation]	1.462	2.117	1.500	1.120	1.979	1.098	1.637	1.007	1.469	1.431	1.633	1.404	1.146
AGAP001314-RA		1.447	2.271	1.519	1.067	1.718	1.802	1.210	1.077	1.025	1.227	1.262	1.834	1.354
AGAP004163-RA	GSTD7	1.434	1.188	1.204	2.086	1.679	2.104	2.019	1.236	1.333	1.371	1.811	1.050	1.466
AGAP010499-RA		1.419	1.374	1.560	1.216	1.202	1.626	0.926	1.220	1.170	2.145	1.707	1.451	1.605
AGAP007059-RA		1.417	1.179	1.258	1.515	1.976	1.409	1.775	1.059	1.242	1.686	1.651	1.061	1.199
AGAP010499-RA		1.417	1.374	1.560	1.216	1.202	1.626	0.926	1.220	1.170	2.145	1.707	1.451	1.605
AGAP003903-RA	Required for meiotic nuclear division 1 homolog (S. cerevisiae) [Source:VB Community Annotation]	1.410	2.253	1.898	1.116	1.337	1.289	1.792	1.249	1.105	1.632	1.000	1.161	1.084
AGAP006364-RA	ABC-B (AGAP006364)	1.401	1.614	1.707	2.360	3.594	2.583	1.075	1.068	1.475	0.936	1.604	1.234	1.390
AGAP010499-RA		1.371	1.374	1.560	1.216	1.202	1.626	0.926	1.220	1.170	2.145	1.707	1.451	1.605
AGAP006617-RA	Armadillo repeat-containing protein 6 [Source:VB Community Annotation]	1.355	1.482	1.475	1.604	1.321	1.483	1.325	1.079	1.059	1.175	1.527	1.099	1.630
AGAP010414-RA	CYP4C28	1.335	1.265	1.250	1.160	1.223	1.147	0.962	1.317	1.507	1.302	1.782	1.624	1.083
AGAP012143-RA	mTERF domain-containing protein, mitochondrial [Source:VB Community Annotation]	1.318	1.184	1.565	1.131	1.155	1.291	1.398	1.181	1.654	1.176	1.015	1.534	1.533
AGAP013519-RA	U6 snRNA-associated Sm-like protein LSm3 [Source:VB Community Annotation]	1.291	1.739	1.033	1.292	1.425	1.055	1.704	1.013	1.220	1.064	1.141	1.684	1.118
AGAP006700-RA	COEAE1G	1.290	1.471	1.319	1.160	1.606	1.368	1.037	1.050	1.041	1.099	1.157	1.546	1.621
AGAP010346-RA	merlin [Source:VB Community Annotation]	1.287	1.216	1.170	1.340	1.696	1.354	1.655	1.149	1.029	1.021	1.004	1.785	1.023
AGAP007564-RA		1.280	1.404	1.227	1.135	1.634	1.014	1.376	1.185	1.028	1.190	1.535	1.085	1.547
AGAP010447-RA	gamma-glutamylcyclotransferase [Source:VB Community Annotation]	1.272	1.204	1.127	1.117	1.371	1.116	1.552	1.534	1.199	1.315	1.143	1.095	1.494
AGAP006884-RA		1.252	1.130	1.133	1.401	1.580	1.180	1.460	1.061	1.190	1.135	1.430	1.219	1.108
AGAP011816-RA		1.247	1.864	1.260	1.169	1.258	1.104	1.088	1.281	1.012	1.106	1.525	1.264	1.027
AGAP005500-RA	dehydrogenase/reductase SDR family member 11 precursor [Source:VB Community Annotation]	1.229	1.530	1.110	1.084	1.100	1.106	1.031	1.045	1.055	1.050	1.067	1.791	1.786
AGAP007395-RA	DIS3-like exonuclease 2 [Source:UniProtKB/TrEMBL;Acc:Q7QJB5]	1.223	1.020	1.193	1.241	1.501	1.183	1.260	1.020	1.303	1.360	1.201	1.094	1.299
AGAP006700-RA	COEAE8O	1.186	1.471	1.319	1.160	1.606	1.368	1.037	1.050	1.041	1.099	1.157	1.546	1.621
AGAP006567-RA		1.175	1.429	1.332	1.251	1.296	1.067	1.086	1.075	1.396	1.008	1.069	1.020	1.070
AGAP004958-RA	ethanolaminephosphotransferase [Source:VB Community Annotation]	1.164	1.168	1.319	1.249	1.106	1.070	1.001	1.111	1.097	1.236	1.009	1.184	1.423
AGAP013143-RA	Rho GTPase-activating protein 26 [Source:VB Community Annotation]	1.083	1.073	1.118	1.029	1.212	1.050	1.078	1.100	1.079	1.014	1.057	1.120	1.063
AGAP006245-RA	zinc finger matrin-type protein 2 [Source:VB Community Annotation]	0.962	0.997	0.911	0.986	0.895	0.928	0.962	0.993	0.991	0.939	0.983	0.994	0.968
AGAP006412-RB		0.951	0.849	0.928	0.955	0.960	0.979	0.972	0.968	0.983	0.996	0.894	0.962	0.964
AGAP003274-RA	solute carrier family 12 (sodium/potassium/chloride transporter), member 2 [Source:VB Community An	0.947	0.906	0.980	0.987	0.953	0.935	0.980	0.990	0.976	0.974	0.940	0.870	0.866
AGAP010593-RA		0.943	0.955	0.905	0.967	0.922	0.999	0.955	0.970	0.999	0.926	0.870	0.953	0.894
AGAP008241-RA	dual-specificity tyrosine-(Y)-phosphorylation regulated kinase [Source:VB Community Annotation]	0.942	0.871	0.910	0.963	0.943	0.962	0.928	0.955	0.990	0.953	0.896	0.949	0.979
AGAP010619-RA	eupolytin [Source:VB Community Annotation]	0.939	0.934	0.954	0.936	0.967	0.914	0.968	0.980	0.959	0.935	0.918	0.860	0.937
AGAP002519-RA	putative tyramine receptor [Source:VB Community Annotation]	0.935	0.997	0.862	0.949	0.891	0.899	0.944	0.980	0.904	0.918	0.982	0.962	0.926
AGAP009003-RA		0.932	0.871	0.882	0.986	0.953	0.950	0.974	0.989	0.970	0.980	0.855	0.855	0.918
AGAP011217-RA	E3 ubiquitin-protein ligase RBBP6 [Source:VB Community Annotation]	0.920	0.893	0.901	0.970	0.945	0.980	0.939	0.928	0.958	0.949	0.810	0.846	0.917
AGAP012257-RA	neuroglobin [Source:VB Community Annotation]	0.919	0.944	0.852	0.990	0.903	0.966	0.934	0.985	0.927	0.885	0.897	0.920	0.825
AGAP005254-RA		0.919	0.859	0.920	0.977	0.938	0.910	0.937	0.930	0.945	0.944	0.982	0.890	0.792
AGAP006133-RA	nucleosome-remodeling factor subunit BPTF [Source:VB Community Annotation]	0.918	0.881	0.970	0.948	0.956	0.911	0.992	0.981	0.977	0.993	0.985	0.701	0.726
AGAP009285-RA		0.903	0.845	0.973	0.963	0.998	0.936	0.953	0.980	0.855	0.783	0.862	0.887	0.797
AGAP000403-RA		0.854	0.657	0.946	0.890	0.984	0.935	0.819	0.889	0.934	0.877	0.910	0.915	0.490
AGAP008433-RA	RNA-binding protein 39 [Source:VB Community Annotation]	0.850	0.796	0.926	0.906	0.835	0.778	0.692	0.904	0.885	0.893	0.923	0.772	0.888
AGAP001565-RA	vacuolar protein sorting-associated protein [Source:VB Community Annotation]	0.849	0.798	0.999	0.822	0.557	0.770	0.760	0.996	0.888	0.994	0.831	0.822	0.950
AGAP006784-RA	F-box protein 9 [Source:VB Community Annotation]	0.848	0.863	0.921	0.811	0.902	0.808	0.804	0.972	0.851	0.984	0.813	0.727	0.722
AGAP001726-RA		0.840	0.951	0.856	0.861	0.784	0.791	0.781	0.913	0.841	0.875	0.883	0.804	0.741
AGAP002986-RA	p21-activated kinase 1 [Source:VB Community Annotation]	0.823	0.893	0.823	0.884	0.700	0.758	0.893	0.994	0.996	0.848	0.649	0.971	0.472
AGAP011545-RA		0.809	0.707	0.771	0.767	0.770	0.733	0.773	0.965	0.945	0.960	0.719	0.609	0.984
AGAP006599-RA	ATP-dependent RNA helicase A [Source:VB Community Annotation]	0.808	0.722	0.775	0.852	0.870	0.943	0.569	0.983	0.720	0.804	0.728	0.919	0.817
AGAP000512-RA	transcriptional enhancer factor [Source:VB Community Annotation]	0.799	0.736	0.979	0.891	0.753	0.878	0.645	0.968	0.931	0.811	0.748	0.567	0.682
AGAP009920-RA	60S ribosomal protein L37a [Source:VB Community Annotation]	0.778	0.837	0.831	0.815	0.624	0.704	0.818	0.865	0.719	0.794	0.736	0.780	0.810
AGAP002110-RA	cell division control protein 7 [Source:VB Community Annotation]	0.717	0.846	0.744	0.597	0.526	0.679	0.491	0.769	0.950	0.951	0.757	0.738	0.552
AGAP000792-RA	Adenosylhomocysteinase [Source:UniProtKB/TrEMBL;Acc:Q7QE71]	0.711	0.446	0.729	0.664	0.473	0.715	0.592	0.957	0.928	0.700	0.885	0.687	0.756
AGAP005187-RA		0.692	0.493	0.572	0.989	0.723	0.818	0.562	0.568	0.862	0.960	0.886	0.638	0.237
AGAP006438-RA	ribosomal biogenesis protein LAS1 [Source:VB Community Annotation]	0.687	0.995	0.277	0.382	0.328	0.361	0.960	0.992	0.992	0.730	0.947	0.947	0.332
AGAP008393-RA	DNA-directed RNA polymerase II subunit RPB9 [Source:VB Community Annotation]	0.680	0.850	0.507	0.578	0.710	0.702	0.832	0.722	0.442	0.547	0.318	0.966	0.983
AGAP007668-RA	Eukaryotic translation initiation factor 3 subunit G [Source:UniProtKB/Swiss-Prot;Acc:Q7QJV0]	0.581	0.236	0.255	0.349	0.421	0.272	0.763	0.927	0.968	0.720	0.822	0.456	0.785
AGAP001161-RA	long wavelength sensitive opsin 6 [Source:VB Community Annotation]	0.403	0.220	0.530	0.245	0.375	0.209	0.921	0.464	0.379	0.602	0.199	0.226	0.461

Supplementary Table 3: Transcripts showing consistently significant fold change directionality across all pyrethroid resistant Anopheles coluzzii transcripts. VectorBase ID, Transcript name or desctiption, the average fold changes across all the arrays and the fold changes for the relevant data sets are displayed. Transcripts consistently up-regulated trend are highlighte in red, and down-regulated in green.

VectorBase ID	Gene Name/Description	Average Fold Change	BiokoFC	BouakeFC	MBeFC	TiassaleFC	VK6FC	BanforaFC	TengrelaFC	VK72012FC	VK72011FC	TiassaleMaliFC	Tiassale2011FC
AGAP006879-RA	F-type H+-transporting ATPase subunit e [Source:VB Community Annotation]	37.262	102.582	7.876	34.885	58.465	13.418	19.793	15.424	33.888	9.524	26.519	87.510
AGAP013192-RA	venom allergen [Source:VB Community Annotation]	29.939	30.696	21.823	52.061	86.939	1.380	19.253	2.363	62.987	3.284	1.586	46.958
AGAP002364-RB	MICOS complex subunit MIC60 [Source:UniProtKB/TrEMBL;Acc:F5HK71]	12.681	18.552	10.760	27.297	25.023	3.206	3.991	1.704	19.588	1.395	1.809	26.169
AGAP002364-RA	MICOS complex subunit MIC60 [Source:UniProtKB/TrEMBL;Acc:F5HK71]	11.958	18.386	9.180	25.921	21.336	3.114	3.924	1.629	19.421	1.374	1.774	25.480
AGAP007160-RB	alpha-crystallin chain B [Source:VB Community Annotation]	11.467	7.841	15.426	14.444	13.709	17.990	34.759	5.672	4.103	2.878	3.796	5.519
AGAP007160-RC	alpha-crystallin chain B [Source:VB Community Annotation]	11.170	7.719	15.190	14.124	13.368	18.747	32.009	5.550	4.047	2.898	3.755	5.464
AGAP007160-RA	alpha-crystallin chain B [Source:VB Community Annotation]	10.095	7.869	14.592	13.541	11.943	14.287	26.791	5.890	4.110	2.912	3.812	5.303
AGAP012982-RA	long wavelength sensitive opsin 3 [Source:VB Community Annotation]	9.388	13.565	2.769	2.610	3.560	15.870	13.664	3.285	11.278	2.077	2.640	31.954
AGAP009110-RA		8.085	5.811	5.266	8.426	5.393	4.127	2.433	21.802	3.330	21.656	7.971	2.721
AGAP008684-RA	juvenile hormone epoxide hydrolase [Source:VB Community Annotation]	5.635	3.940	4.022	3.649	4.468	3.094	13.781	4.654	8.069	5.753	3.002	7.554
AGAP006383-RA	oligosaccharyltransferase complex subunit beta [Source:VB Community Annotation]	5.009	9.396	2.542	5.680	8.040	1.360	1.682	3.496	7.030	4.288	4.155	7.426
AGAP012608-RA	Proteasome subunit beta type [Source:UniProtKB/TrEMBL;Acc:Q7QLL8]	4.971	9.759	2.236	2.096	2.286	2.929	9.342	1.831	9.722	2.014	1.633	10.828
AGAP007249-RB		4.760	7.461	2.588	2.845	1.897	2.492	10.802	1.768	9.017	1.500	2.035	9.958
AGAP009828-RA		4.730	2.328	6.824	3.268	6.985	7.619	7.098	2.276	4.101	2.029	2.032	7.468
AGAP008438-RA		4.580	2.136	3.803	4.237	2.248	3.054	20.397	2.691	4.877	2.546	1.495	2.891
AGAP001136-RA	tetratricopeptide repeat protein 35 [Source:VB Community Annotation]	4.498	2.329	2.968	8.428	5.736	1.601	4.176	4.497	6.309	5.442	3.020	4.975
AGAP000513-RA	dipeptidase E [Source:VB Community Annotation]	4.085	16.017	3.224	2.738	1.700	1.527	2.977	2.191	7.782	2.538	1.899	2.347
AGAP001998-RA	28S ribosomal protein S10, mitochondrial [Source:VB Community Annotation]	4.033	9.593	2.709	2.595	2.181	1.783	5.304	2.105	9.582	1.734	1.360	5.420
AGAP007780-RA	NADH dehydrogenase (ubiquinone) 1 beta subcomplex 10 [Source:VB Community Annotation]	3.638	4.439	5.378	3.410	2.545	1.385	6.942	3.907	2.989	1.509	2.918	4.599
AGAP011052-RA	aldose reductase [Source:VB Community Annotation]	3.473	3.520	2.464	4.328	5.404	1.258	4.371	3.330	5.772	3.192	2.044	2.526
AGAP007249-RA		3.257	1.837	2.059	3.908	4.037	1.445	2.505	3.217	6.356	3.145	2.948	4.369
AGAP002940-RA	coiled-coil-helix-coiled-coil-helix domain containing 4 [Source:VB Community Annotation]	2.893	3.939	2.658	2.495	2.964	1.183	3.314	3.713	2.428	3.203	2.710	3.220
AGAP007249-RA		2.873	1.837	2.059	3.908	4.037	1.445	2.505	3.217	6.356	3.145	2.948	4.369
AGAP002419-RA	CYP4D22	2.760	3.102	3.009	3.449	4.124	1.659	2.034	2.112	2.047	2.262	2.197	3.959
AGAP007403-RA		2.306	1.690	2.058	2.497	2.078	4.604	1.872	1.997	1.700	2.053	2.165	2.651
AGAP007589-RA	UGT306A2	2.272	1.821	1.305	1.808	3.458	1.557	1.688	2.677	2.754	3.090	1.955	2.879
AGAP006651-RA	peroxisomal 3,2-trans-enoyl-CoA isomerase [Source:VB Community Annotation]	2.160	2.474	1.283	1.201	1.163	2.959	2.210	2.856	2.718	2.358	2.161	2.373
AGAP012953-RA	epidermal retinal dehydrogenase [Source:VB Community Annotation]	2.084	2.232	2.453	1.742	3.519	1.655	2.478	2.144	1.746	1.711	1.596	1.646
AGAP013502-RA		1.806	2.105	1.590	1.732	1.651	1.928	2.260	1.166	2.206	1.275	1.241	2.709
AGAP007115-RA		0.642	0.863	0.700	0.739	0.527	0.675	0.339	0.666	0.619	0.838	0.542	0.552
AGAP007120-RA	nucleoside-diphosphate kinase [Source:VB Community Annotation]	0.610	0.800	0.362	0.487	0.395	0.495	0.639	0.815	0.698	0.719	0.796	0.505
AGAP012788-RA	cytosolic Fe-S cluster assembly factor NUBP2 homolog [Source:VB Community Annotation]	0.609	0.788	0.547	0.527	0.564	0.507	0.496	0.698	0.490	0.759	0.809	0.509
AGAP002101-RA	isoleucyl-tRNA synthetase [Source:VB Community Annotation]	0.568	0.469	0.635	0.805	0.520	0.650	0.591	0.512	0.586	0.514	0.528	0.443
AGAP003485-RA	thioredoxin domain-containing protein [Source:VB Community Annotation]	0.481	0.711	0.292	0.326	0.408	0.550	0.371	0.440	0.539	0.558	0.656	0.442
AGAP002969-RA	asparaginyl-tRNA synthetase [Source:VB Community Annotation]	0.475	0.662	0.448	0.657	0.384	0.536	0.493	0.422	0.400	0.457	0.423	0.342
AGAP004684-RA	rRNA-processing protein CGR1 [Source:VB Community Annotation]	0.307	0.140	0.121	0.119	0.277	0.609	0.580	0.439	0.215	0.274	0.356	0.244
AGAP004880-RC	L-lactate dehydrogenase [Source:UniProtKB/TrEMBL;Acc:Q7Q981]	0.259	0.198	0.250	0.403	0.174	0.497	0.161	0.112	0.296	0.284	0.213	0.265
AGAP004880-RA	L-lactate dehydrogenase [Source:UniProtKB/TrEMBL;Acc:Q7Q981]	0.251	0.202	0.249	0.382	0.180	0.413	0.179	0.112	0.305	0.278	0.214	0.252
AGAP004880-RB	L-lactate dehydrogenase [Source:UniProtKB/TrEMBL;Acc:Q7Q981]	0.246	0.194	0.243	0.394	0.181	0.362	0.175	0.109	0.294	0.277	0.214	0.263
AGAP006414-RA	chitinase [Source:VB Community Annotation]	0.212	0.391	0.254	0.280	0.456	0.063	0.016	0.027	0.012	0.012	0.488	0.334

Supplementary Table 4: Probe and dsRNA construct BLAST. (Sheet 1: Microarray Probes): Gene ID, Gene Name, Probe Sequence, Anopheles gambiae BLAST hits, % identity of off target BLAST hits and the alignment of the off target BLAST hits. Highlighted in yellow are probes with potential cross-hybridisation due to the similarity of the off-target sequences (Sheet 2: dsRNA): Gene ID, Gene Name, Construct Sequence, Anopheles gambiae BLAST hits, % identity of off target BLAST hits, and information on the construct alignment and putative off-target siRNA targets. Highlighted in yellow are constructs that have at least 1 off-target hit which align with 100% identity to a 20bp siRNA, which may be produced from the dsRNA in vivo.

<table>
<tr><th>ID</th><th>Name</th><th>Probe</th><th>Off-Target Significant BLAST hits (>55/60 alignment length)</th><th>Identity to off-target</th><th>Alignment</th></tr>
<tr><td>AGAP004164</td><td>GSTD1</td><td>GACTTCAGTGCGTATGTAAATGTTCTGCGATGGTATAAGAGCATGCCCGAATTGATTCCC</td><td>NONE</td><td></td><td></td></tr>
<tr><td>AGAP004163</td><td>GSTD7</td><td>TAATTGCTCCCATTTGTCGTTGTACGCTAGTGTATAGGCAGTTTGTTATTCGAAGCCTTT</td><td>NONE</td><td></td><td></td></tr>
<tr><td>AGAP004382</td><td>GSTD3</td><td>TAAGGGATTGAAAGTGTAACTGCATTAGCAACACCTCAAGTGGTAGACTATTGTGTCAGG</td><td>NONE</td><td></td><td></td></tr>
<tr><td>AGAP009192</td><td>GSTE5</td><td>TTTGCAGAAATCTGTGAATCGCTGAGGCTTAAAAATGGGGCGTCGGTCGCTGCGAAGTAA</td><td>NONE</td><td></td><td></td></tr>
<tr><td>AGAP009946</td><td>GSTMS3</td><td>GAATAAAGCTTGCTCCTCTATGCATACGGAGAACTGCCACTGGTTCTTTGGGTGTCAAAA</td><td>NONE</td><td></td><td></td></tr>
<tr><td>AGAP006700</td><td>COEAE8O</td><td>ATTACGAACGACGGTATTGCGATGGCCGAGATGCCCGAAAAGCGGCGGATGAACTTTTGG</td><td>NONE</td><td></td><td></td></tr>
<tr><td>AGAP010414</td><td>CYP4C28</td><td>GGCGATTTGGTTATGCGTACCAAAGGCGGTCTTAAGATACGCATCCAAAGAAGAATATAG</td><td>NONE</td><td></td><td></td></tr>
<tr><td>AGAP008020</td><td>CYP12F2</td><td>AAACCCGGTAATGATGCAACCGAAAACAATTAAACTTTACGTCGATCAGGTGGATGAAAT</td><td>AGAP012800</td><td>98.33%</td><td>Query 1 AAACCCGGTAATGATGCAACCGAAAACAATTAAACTTTACGTCGATCAGGTGGATGAAAT 60
||||||||| ||||||||||||||||||||||||||||||||||||||||||||||||||
Sbjct 480 AAACCCGGTGATGATGCAACCGAAAACAATTAAACTTTACGTCGATCAGGTGGATGAAAT 539</td></tr>
<tr><td>AGAP002419</td><td>CYP4D22</td><td>TTGGGAAGCGATGTGAGCGCAAGCGTTGTTTTAGTTATGGGCATAAAAATAAATAATTTG</td><td>NONE</td><td></td><td></td></tr>
<tr><td>AGAP000818</td><td>CYP9K1</td><td>TAACTGCATCGGTTCGCGGTTCGCGCTGATGGAGACGAAGGCGGTGTTTTTCTTCCTGCT</td><td>NONE</td><td></td><td></td></tr>
<tr><td>AGAP001076</td><td>CYP4G16</td><td>CGAAGACATTATGAGGATTGGACGTTTACAGAGCATGCAAACTTCATTGTTATATTATAC</td><td>NONE</td><td></td><td></td></tr>
<tr><td>AGAP000877</td><td>CYP4G17</td><td>AATGTGTGACATCCTGCACAAGCGCCAACTGAAGATCCACCTGCGCCTGGACCCACTGTT</td><td>NONE</td><td></td><td></td></tr>
<tr><td>AGAP008219</td><td>CYP6Z1</td><td>TTAACTTTTCGGCCACCATTCCGAGGAAGATAAAGTTTGAGCCCGTTTCAATCACACTGG</td><td>NONE</td><td></td><td></td></tr>
<tr><td>AGAP008218</td><td>CYP6Z2</td><td>TGAACTTTCGCACAATCCAGAAGCGATGGCAAAGCTGCAGCAAGAAATTGACGAGATGAT</td><td>CYP6Z3</td><td>98.30%</td><td>Query 1 TGAACTTTCGCACAATCCAGAAGCGATGGCAAAGCTGCAGCAAGAAATTGACGAGATGAT 60
|||||||||||||||||||||||| |||||||||||||||||||||||||||||||||||
Sbjct 1012 TGAACTTTCGCACAATCCAGAAGCAATGGCAAAGCTGCAGCAAGAAATTGACGAGATGAT 1071</td></tr>
<tr><td>AGAP008217</td><td>CYP6Z3</td><td>AAGTACAATTTCCAAGCCACAACTCCTGCTAAGGTCAAATTTGCTGTTGCTACTGTAGTT</td><td>CYP6Z2</td><td>82.80%</td><td>Query 1 AAGTACAATTTCCAAGCCACAACTCCTGCTAAGGTCAAATTTGCTGTTGCTACTGTAG 58
|||| ||| ||||| |||||||||||||| || || || ||||| | ||||||||||
Sbjct 1475 AAGTTCAAGTTCCAGGCCACAACTCCTGCAAAAGTAAAGTTTGCAGCCGCTACTGTAG 1532</td></tr>
<tr><td>AGAP008212</td><td>CYP6M2</td><td>AAATTCTTAATGAATCACTGCGCAAGTATCCACCAGTCCCGGTACATTTCCGTGTGGCTT</td><td>NONE</td><td></td><td></td></tr>
<tr><td>AGAP002869</td><td>CYP6P2</td><td>AGCAAGTTTACGCTCAAAATTACCGACGCCGATGTGGAAACGTTCTTCATGAATCTGGTG</td><td>NONE</td><td></td><td></td></tr>
<tr><td>AGAP002865</td><td>CYP6P3</td><td>CCGGGGTTTATAATTTTGTGTTGCAATAAAAGCAAAACAAAGCATCGCTAACATAGAGGC</td><td>NONE</td><td></td><td></td></tr>
<tr><td>AGAP002867</td><td>CYP6P4</td><td>CCAAAGTCCTTCATATTATCACCGTCCACCGGTAATTATCTGAAGGTTGATAAGATATAG</td><td>NONE</td><td></td><td></td></tr>
<tr><td>AGAP012296</td><td>CYP9J5</td><td>GCTAACTGATCCTACTACATAAGATCTGTTAGGAAGCAAGCCTATTGTAGATATTAATCA</td><td>NONE</td><td></td><td></td></tr>
<tr><td>AGAP007158</td><td>a-crystallin</td><td>CATTGATTAGCCTATAGACTCTGTTCAGACTGATGCGAATTTTGCTTTTATTTAGACTGT</td><td>AGAP007159</td><td>100%</td><td>SAME PROBE</td></tr>
<tr><td>AGAP007159</td><td>a-crystallin</td><td>CATTGATTAGCCTATAGACTCTGTTCAGACTGATGCGAATTTTGCTTTTATTTAGACTGT</td><td>AGAP007158</td><td>100%</td><td>SAME PROBE</td></tr>
<tr><td>AGAP007160</td><td>a-crystallin</td><td>GAAATCGTCGTCATTCCGTTTACGCAAGGAGCTAGACTATAGTCTAGTTCGGTTGTGTTT</td><td>NONE</td><td></td><td></td></tr>
<tr><td>AGAP007161</td><td>a-crystallin</td><td>GCGGTGAGAGTGTTCGTGCAAAGGATGCGAAATAAAGTGAAGAATATATTGTTGAGCATT</td><td>NONE</td><td></td><td></td></tr>
<tr><td>AGAP007162</td><td>a-crystallin</td><td>CCGGTCTACCGGTTCCTGAACTGATCGTACAATAAATAAAAAAGAGCTCTTTTATATTGA</td><td>NONE</td><td></td><td></td></tr>
<tr><td>AGAP001345</td><td>Hexamerin</td><td>TGTGCCGAACGCGTTCTTCCAGGACGTCAGCATCTATCACAAGTCCGAGGGTGAGCTGTA</td><td>NONE</td><td></td><td></td></tr>
<tr><td>AGAP001657</td><td>Hexamerin</td><td>TACTACCTCGATACGATGATCTTCCACAAGAAGGAGGCGGAAATCAATGCTGTTCACTAA</td><td>NONE</td><td></td><td></td></tr>
<tr><td>AGAP001659</td><td>Hexamerin</td><td>TACTACGACACGATGATCTTCCACAAGAGCGAAACGGAGGTCAACTCGGTCGTTGTCTAG</td><td>NONE</td><td></td><td></td></tr>
<tr><td>AGAP005766</td><td>Hexamerin</td><td>TACTTCAAGGATGTGTTCATCTTCCACACCGAAGAGATGAAAATGAACCAGACCTACTAA</td><td>AGAP005767</td><td>100%</td><td>SAME PROBE</td></tr>
<tr><td>AGAP005767</td><td>Hexamerin</td><td>TACTTCAAGGATGTCTTCATCTTCCACAATGATGAGATGAAAATGAACCAGACCTACTAA</td><td>AGAP005766</td><td>100%</td><td>SAME PROBE</td></tr>
<tr><td>AGAP005768</td><td>Hexamerin</td><td>AAGAACATGTACTTCAAGGACGTGTTGATCTATCACAACGACGACTTTAAGAAGATGTAC</td><td>NONE</td><td></td><td></td></tr>
<tr><td>AGAP010657</td><td>Hexamerin</td><td>TGTGAACTTCCAAGAAGTGGTTGAGTTCTTCAACTACTACAAGGCTGGATTCCTGGGCAA</td><td>NONE</td><td></td><td></td></tr>
<tr><td>AGAP010658</td><td>Hexamerin</td><td>AGCTGTACCGATTTTTCGAGAATGCGATCGACTGGGATACGTACTACAAGAATGTGGTCT</td><td>NONE</td><td></td><td></td></tr>
<tr><td>AGAP013061</td><td>Unknown</td><td>CACCGGAAACGAATAGGATCGAAATGGTTAAAGACGGAGGAAAACTTATGTTGTGGTTTT</td><td>NONE</td><td></td><td></td></tr>
<tr><td>AGAP002603</td><td>EF-like Factor</td><td>ATCCGTTCCGTTTCAGTGCGTGAGTGTGTGTGAGGAGAAATAAAAGCATAATCAAATCAG</td><td>NONE</td><td></td><td></td></tr>
<tr><td>AGAP008438</td><td>Unknown</td><td>AGCCGAGGTAAACGAGGATCACGAATGTTCAATGTTTGAATTTTTAAAGAAACGAAACAA</td><td>NONE</td><td></td><td></td></tr>
<tr><td>AGAP006879</td><td>ATPase</td><td>AGGGTGTGTGAGGGATAAAAGCATCATCCTGTTCATTTAGAGAAGTAGAATATAAATCGA</td><td>NONE</td><td></td><td></td></tr>
</table>

Supplementary Table 5: VectorBase ID, Gene Description, Log fold change and adjusted p value for transcripts significantly differentially expressed in the Maf-S knockdown arrays compared to GFP-injcted controls. Only transcripts overlapping with transcript list in Supplementary Tables 2 and 3 in this manuscript are shown. The full data set is avaialbe in Ingham et al, 2017.

Maf-S regulated genes overlapping with Table S2

VectorBase ID	Gene Description	logFC	adj.P.Val
AGAP001161-RA	Long Wavelength Sensitive Opsin	2.523	0.002
AGAP002603-RA	Elongation Factor-like	0.743	0.002
AGAP013519-RA	U6 snRNA-associated sm-like Protein	-0.562	0.003
AGAP004113-RA	39S Ribosomal Protein L55	-0.535	0.005
AGAP010499-RA	S-glutathione Dehydrogenase	-0.545	0.005
AGAP002110-RA	Cell Division Control Protein 7	0.705	0.006
AGAP001314-RA		-0.412	0.007
AGAP001659-RA	Hexamerin	0.358	0.008
AGAP006599-RA	ATP-dependent RNA Helicase A	0.460	0.011
AGAP010346-RA	Merlin	-0.556	0.015
AGAP009192-RA	GSTE5	-0.409	0.018
AGAP005187-RA		0.322	0.020
AGAP007161-RA	a-crystallin Chain A	0.688	0.020
AGAP013008-RA		-0.604	0.031
AGAP000512-RA	Transcriptional Enhancer Factor	0.241	0.037
AGAP004958-RA	Ethanolaminephosphotransferase	-0.454	0.038
AGAP007366-RB		0.362	0.045
AGAP008217-RA	CYP6Z3	-0.217	0.049

Maf-S regulated genes overlapping with Table S3

AGAP004880-RC	L-Lactate Dehydrogenase	1.620	0.001
AGAP004880-RB	L-Lactate Dehydrogenase	1.670	0.001
AGAP004880-RA	L-Lactate Dehydrogenase	1.690	0.001
AGAP012982-RA	Long Wavelength Sensitive Opsin	1.040	0.001
AGAP004684-RA	rRNA-processing protein CGR1	-0.865	0.002
AGAP012953-RA	Epidermal retinal dehydrogenase	-0.617	0.002
AGAP007403-RA		0.833	0.005
AGAP007120-RA		-0.733	0.005
AGAP007249-RA		0.575	0.007
AGAP002969-RA	Asparaginyl-tRNA synthetase	0.485	0.010
AGAP009110-RA		-1.520	0.011
AGAP007160-RC	alpha-crystallin chain B	1.380	0.014
AGAP002101-RA	isoleucyl-tRNA synthetase	0.495	0.016
AGAP007160-RB		1.350	0.016
AGAP007249-RB		0.365	0.018
AGAP007160-RA	alpha-crystallin chain B	1.240	0.021

AGAP012788-RA	Cytosolic Fe-S cluster assembly factor NUBP2 homolog	-0.348	0.023
AGAP013192-RA	Venom Allergen	-0.493	0.046

Supplementary Table 6: Detoxification Transcripts showing significantly reduced fold change in dsMet knockdown arrays compared to GFP-injected controls. VectorBase ID, Transcript name in addition to the fold changes across all the arrays are displayed.

VectorBase ID	Gene Name	Fold Change
AGAP011507-RA	COE13O	0.443
AGAP006724-RA	COEAE3G	0.714
AGAP005372-RA	COEBE3C	0.678
AGAP005658-RA	CYP15B1	0.776
AGAP005992-RA	CYP302A1	0.58
AGAP008682-RA	CYP307B1	0.769
AGAP002429-RA	CYP314A1	0.796
AGAP009246-RA	CYP4C27	0.579
AGAP009241-RA	CYP4C36	0.706
AGAP002418-RA	CYP4D16	0.731
AGAP000877-RA	CYP4G17	0.646
AGAP001864-RA	CYP4H15	0.609
AGAP002416-RA	CYP4K2	0.546
AGAP008212-RA	CYP6M2	0.375
AGAP008213-RA	CYP6M3	0.332
AGAP008208-RA	CYP6Y1	0.559
AGAP008207-RA	CYP6Y2	0.584
AGAP012294-RA	CYP9L2	0.642
AGAP004172-RA	GSTD9	0.717
AGAP009195-RA	GSTE1	0.761
AGAP009197-RA	GSTE3	0.608
AGAP009191-RA	GSTE6	0.794
AGAP000165-RA	GSTMS1	0.779
AGAP008404-RA	UGT301C1	0.645
AGAP007028-RA	UGT302J1	0.652
AGAP005750-RA	UGT308B2	0.546
AGAP007374-RA	UGT49A3	0.526

Supplementary Table 7: Enrichment in Tiassale abdomen carcass of transcripts represented in the CYP4G16 correlation network. ID, Log2 Fold change, adjusted p value and absolute fold change of all transcripts present in the CYP4G16 correlation network at cut-of p=0.85. Highlighted in blue are significant (p<=0.05) adjusted p values, red show enriched transcripts and green down-regulated transcripts in the abdomen carcass of Tiassale. See Ingham et al. 2014

SystematicName	logFC	adj.P.Val	FC
AGAP013094-RA	3.176254258	0.000147	9.039571
AGAP001899-RA	3.171054945	8.17E-05	9.007052
AGAP000877-RA	2.73208152	8.70E-05	6.644136
AGAP003197-RA	2.72357561	5.83E-05	6.605078
AGAP001473-RA	2.705907289	0.000156	6.524681
AGAP010059-RA	2.688152585	9.38E-05	6.444876
AGAP009609-RA	2.562919543	0.000192	5.909023
AGAP001076-RA	2.560553916	3.73E-05	5.899341
AGAP001076-RB	2.513719311	5.29E-05	5.710905
AGAP001076-RC	2.505468505	5.83E-05	5.678337
AGAP012405-RA	2.186076912	0.000114	4.550664
AGAP005984-RA	2.126856101	0.000155	4.367647
AGAP003239-RA	2.105140554	0.000489	4.302397
AGAP003733-RA	2.09288528	9.38E-05	4.266004
AGAP005984-RB	2.089960943	0.000211	4.257365
AGAP001076-RD	2.069975002	9.64E-05	4.198794
AGAP003050-RA	2.020409464	0.00041	4.056989
AGAP007692-RA	1.96967687	9.32E-05	3.916804
AGAP004318-RA	1.850045116	0.000143	3.605115
AGAP008012-RA	1.803679004	0.000186	3.491094
AGAP009874-RA	1.580966153	0.003443	2.991701
AGAP002925-RC	1.563619162	5.84E-05	2.955944
AGAP003571-RC	1.560666445	0.001812	2.949901
AGAP002925-RA	1.550844523	5.83E-05	2.929886
AGAP010193-RA	1.541293971	0.000653	2.910554
AGAP002925-RB	1.540051014	5.84E-05	2.908048
AGAP001181-RA	1.505647405	0.000369	2.839521
AGAP004719-RA	1.48767276	8.38E-05	2.804362
AGAP009879-RA	1.353087829	0.000997	2.554583
AGAP007939-RA	1.312314519	0.001418	2.483396
AGAP006410-RA	1.081436531	0.00023	2.116142
AGAP006410-RB	1.058175994	0.000266	2.082297
AGAP003168-RA	1.007105755	0.000882	2.009875
AGAP010325-RA	0.925666085	0.000576	1.899561
AGAP013507-RA	0.599688705	0.003968	1.51539
AGAP007119-RA	0.2545319	0.340874	1.192949
AGAP001903-RA	-0.1776637	0.436704	0.884134
AGAP004992-RA	-0.497289689	0.004022	0.708436
AGAP010226-RA	-0.534951529	0.015484	0.690182
AGAP004722-RA	-0.595734568	0.027057	0.661707
AGAP007827-RA	-0.62765219	0.133673	0.647229
AGAP002500-RA	-0.694479005	0.007311	0.617932
AGAP004778-RA	-0.876842047	0.001298	0.544558
AGAP010517-RA	-0.896942993	0.002295	0.537023

Supplementary Table 8: qPCR primer sets and dsRNA primer sets. (a) qPCR Primers: AGAP identifier, gene description, forward and reverse primers, product size and the number of splice variants the primer covers. (b) RNAi primers: AGAP identifier, gene description, forward and reverse primers and product size

ID	Info	Forward Primer	Reverse Primer	Size	Splice variants
AGAP008438	Unknown	TTAAGCCACCAGAGGGCAAC	TCCAACTCGTTCCCCAACTC	103	1
AGAP006879	ATPase	GTTCCTGGTCGTCGGAGTTG	TGGCGTCACGGATAACCTTC	110	1
AGAP013061	JH-related	AGGATCATCTGTCCCTCGC	TGATTTCACCCAGCACACGG	105	1
AGAP002603	EF-like protein	AAAACCATCCCAGCAAACGTG	TCGTAATCATCGTAGACAGCGT	137	1
AGAP007160	alpha-crystallin chain B	AGCGGTTCAACTTTTTTCCTCC	AAGCGTTGTTGATCGAATCTCT	155	4
AGAP007161	alpha-crystallin chain A	GAACGAACCGTCCCGATACA	TCTGTCCGCTCATTTGCCAT	105	1
AGAP007162	alpha-crystallin chain A	ACACTTTACACGCCGCTACA	GACTTTTTCGGTGCGGTCAC	105	2
AGAP007159	alpha-crystallin chain B	GCACTGTGAGTTCATCAGTTGAATA	TGCCGCTTGAGAAATGTCCT	105	1
AGAP007158	alpha-crystallin chain B	AGACACTTTGTGCGACGCTA	TGATGGGGATGCTTCGTTCC	145	1
AGAP001659	Hexamerin	TACAGTGCGTTCATCCCGAG	CAGGCCAATGTCCTCCATGA	93	1
AGAP001345	Hexamerin	GGGTGAAGTGTTCTCCGTGT	TCGTTGACGTAGTAGCGAGC	132	1
AGAP001657	Hexamerin	TACAAGTGGGTGATGGACGC	TGAAGTACAGCTGGAACGGC	136	1
AGAP005766	Hexamerin	AACCACAAGCCGTTCAGCTA	AAACTTGGGTCCGACGAACG	87	1
AGAP005767	Hexamerin	AGACCTACGCTGTGTTCACC	GAACTTGGGTCCGACAAACG	128	1
AGAP005768	Hexamerin	ATACGCCGACAAGGATTTGCTG	TGGACTCATCCGACACCCAG	124	1
AGAP000646	Dm	CCGTCCGATTCCGATGAAGAAATC	ACGAGGCGGTGCGTATCTT	127	1
AGAP006022	Met	TGCGGGTGGACTATGTGTTT	ACGTCACCGTGAGCAGAAAT	108	1

Supplementary Note 1: IR-TEx User Guide

User Guide

IR-TEx: Insecticide Resistance Transcript Explorer

V.A Ingham, D. Peng, S. Wagstaff and H. Ranson

LSTM
LIVERPOOL SCHOOL
OF TROPICAL MEDICINE

Contents

Section 1: Introduction 1
IR-TEx basics 1
Performance and Resources 2
Installing IR-TEx 2
Section 2: Inputting other resistance datasets 3
Overview of Entering Data 3
Adding data to the web-based app 3
Adding data to a local IR-TEx installation 3
Section 3: Adapting the App to Handle Other Expression Data 5
Creating a new data file 5
Populating the data file 5
Modifying the R code to accept your data 6
Use of one colour arrays and RNASeq data with the app 6
Section 4: References 9
Where to find help 9
Version 9

Section 1: Introduction

IR-TEx is an app written in ShinyR to explore microarray datasets that compare resistant and susceptible *Anopheles gambiae*, *An. coluzzi* and *An. arabiensis* populations, available in public repositories, in a used friendly manner. In its current form, IR-TEx allows the user to search for transcripts of interest using a VectorBase Transcript ID by: Country; Exposure Status; Species and Insecticide Class. The user can also find co-correlated transcripts across experiments by manipulating the Absolute Correlation Value (recommended: 0.7-0.9). The outputs from IR-TEx come in several forms.

IR-TEx basics

IR-TEx can be used to explore the relationships between expression levels of transcripts across populations of Anopheline vectors with varying levels of resistance to insecticides. To run the IR-TEx simply visit the GitHub page which will contain a link to the current application web page. IR-TEx is currently hosted at: https://www.lstmed.ac.uk/projects/ir-tex

The output **below** shows the appearance of a typical ***Interactive Dashboard*** displaying transcript expression, experiment and geographical location.

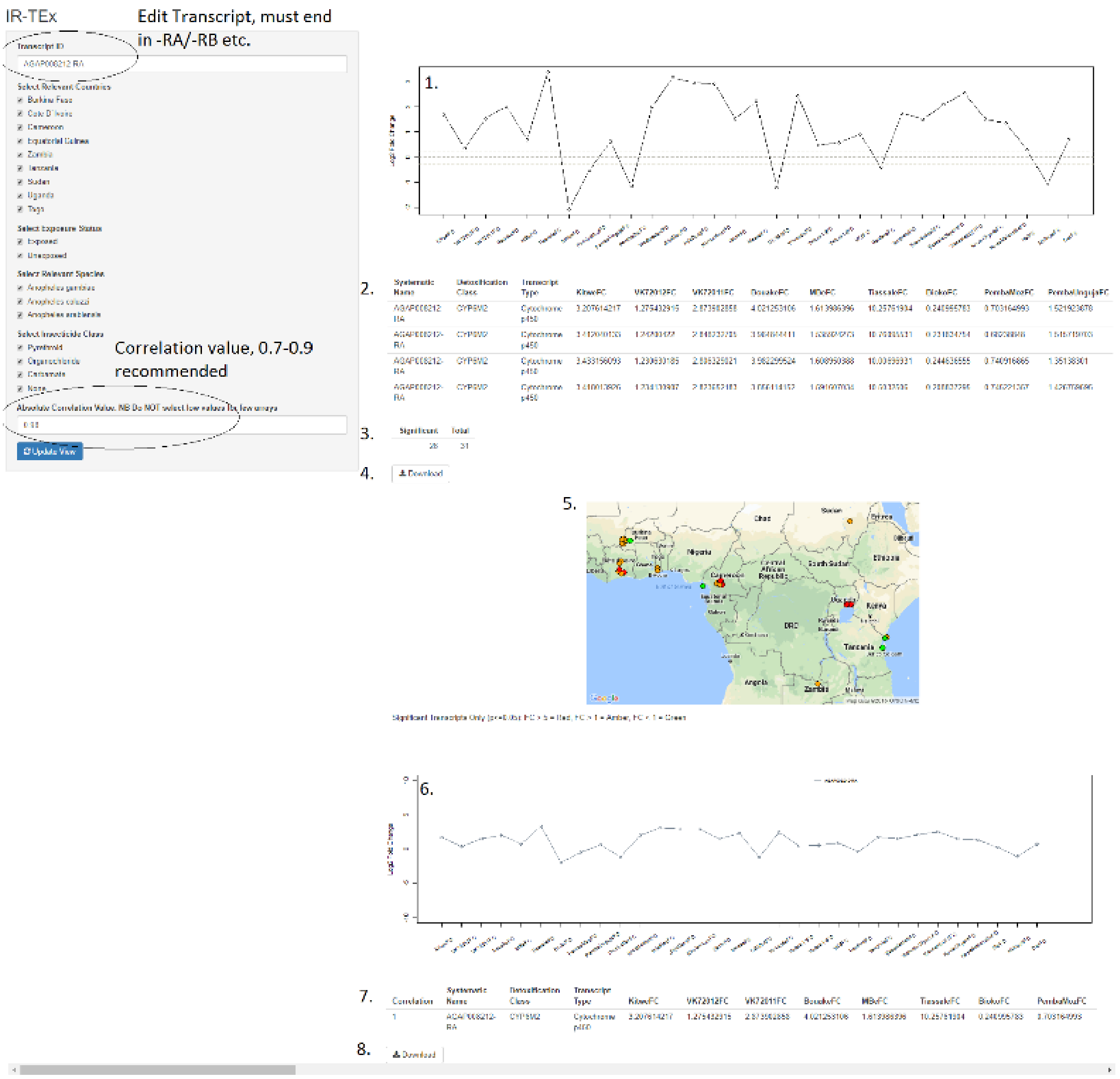

The interactive dashboard is composed of the following:

1) ***Expression Line Graph*** showing the $\log_2$ fold change of the transcript of interest for each microarray data set
2) ***Probe Expression Table*** showing the VectorBase Transcript ID, Detoxification Class, Transcript Description, raw Fold Change (FC) and Q value (Q) (adjusted p-value) for each **probe** (row) and dataset (column).
3) ***Summary Data*** showing the number of arrays in which the transcript is significantly differential.
4) ***Download*** to obtain a local copy of the Probe Expression Table.
5) ***Map*** highlighting the location of the data set containing the transcript of interest with significant differential expression illustrated as a traffic light system.
6) ***Correlation Line Graph*** showing the $\log_2$ fold change of the transcript of interest and transcripts correlated with the transcript of interest (if any) with an absolute correlation greater than that user defined threshold.
7) ***Correlated Transcript Expression Table*** showing the VectorBase Transcript ID, Detoxification Class, Transcript Description, raw Fold Change (FC) and Q value (Q) (adjusted p-value) for each **transcript of interest or correlated transcript** (row) and dataset (column).
8) ***Download*** to obtain a local copy of the Correlated Transcript Expression Table

Performance and Resources

IR-TEx requires only a relatively modest amount of computing power per user. The most computationally intensive part of the application is the initial generation of the correlation matrix. The default matrix that loads on application startup includes an optimum number of datasets and correlation threshold which typically takes ~4s to load on standard Intel i7 processor, consuming ~3GB of RAM in the process. IR-TEx is best ran locally if regular use is intended.

NB. Recalculation of the matrix is required each time a dataset is added or subtracted or an option changed. An increased amount of processing power is required if, for example, all studies are included or a low correlation threshold is selected.

Installing IR-TEx

IR-TEx is a ShinyR application and can be downloaded and installed for free from the following GitHub site at https://github.com/LSTMScientificComputing/IR-TEx and includes a number of files, including a table of fold change and Q values, and a longitude-latitude file for geographical locations of the collection sites. To install locally, the app needs to be installed alongside the packages dismo (https://cran.r-project.org/web/packages/dismo/index.html), rgdal (https://cran.r-project.org/web/packages/rgdal/index.html), shinycssloaders (https://cran.r-project.org/web/packages/shinycssloaders/index.html) and ShinyR. Instructions for the latter are available here - https://shiny.rstudio.com.

Section 2: Inputting other resistance datasets

Overview of Entering Data

All datasets used in this app are currently from the LSTM Agilent 15K array V1 (A-MEXP-2196), dating from AgamP3.5 (2009). Although this array is the most commonly used for insecticide resistance experiments due to the multiple probes for 'detoxification family' genes, we recognise that other array designs may be used in the future. There are two options for inputting these datasets; the first is to use fold changes and Q values for probes only found on the original arrays and the second is to set missing probes to '0' which would cause them to be missing within the app. Below is a walkthrough to adding more resistance datasets to the existing data, without having to change any core code within the app.

Adding data to the web-based app

To add new datasets to the existing web-based app, please email the first author with the new experimental files and designs, in addition to latitude and longitude of collection site of the resistant population: victoria.ingham@lstmed.ac.uk.

Adding data to a local IR-TEx installation

The following is a step-by-step guide to adding data to a **local installation** of IR-TEx. Please follow the steps below

1. Get the data files - within the github repository, there are a number of files, including the user guide. Please download the following files and save then to an appropriate folder: Fold Changes.txt, geography.txt, IR-TEx.R.
2. Open the file Fold Changes.txt – this is a large excel file with RAW fold changes (NOT logged).
3. Examine the file - The first row contains the name of the population, followed by FC (necessary) and also by Q (necessary). Find the final column with FC and insert a row to the right, as illustrated below:

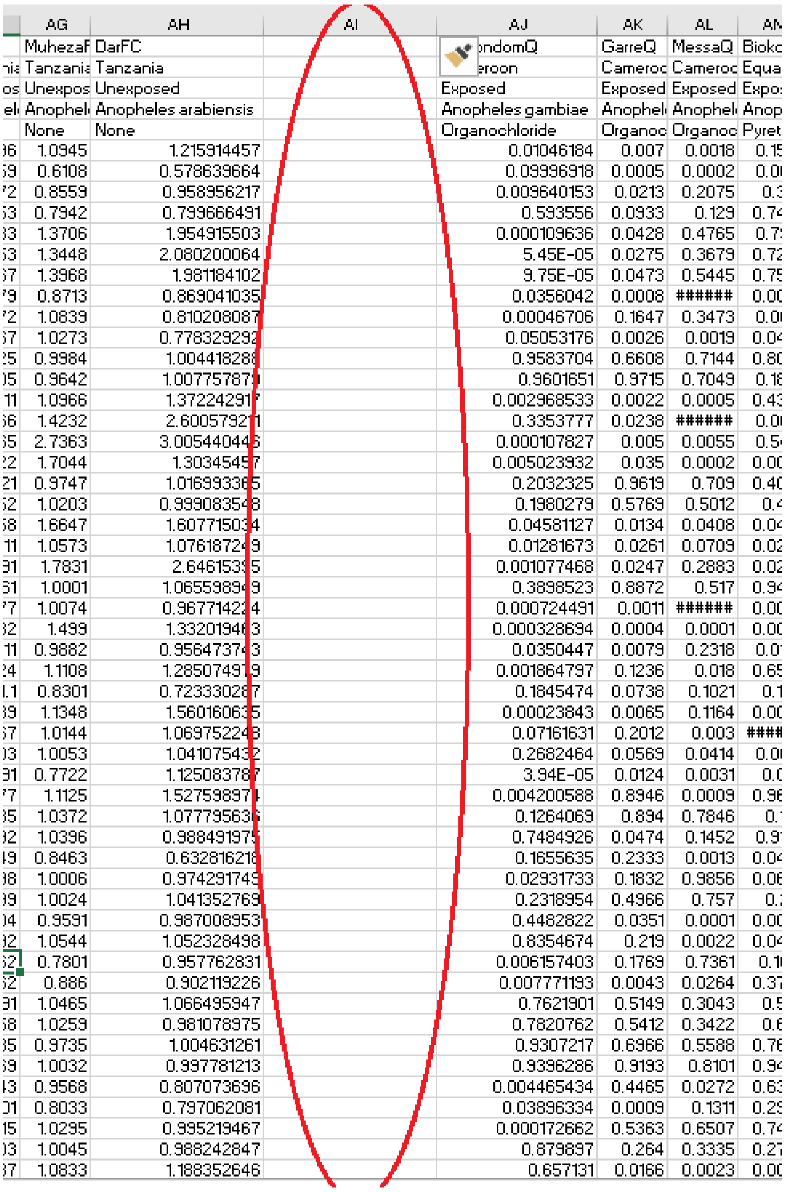

AG	AH	AI	AJ	AK	AL	AM
MuhezaF	DarFC		ndomQ	GarreQ	MessaQ	Biokc
Tanzania	Tanzania		eroon	Cameroc	Cameroc	Equa
Unexpos	Unexposed		Exposed	Exposed	Exposed	Expo:
Anophel	Anopheles arabiensis		Anopheles gambiae	Anophel	Anophel	Anop
None	None		Organochloride	Organoc	Organoc	Pyret
1.0945	1.215914457		0.01046184	0.007	0.0018	0.15
0.6108	0.578639664		0.09996918	0.0005	0.0002	0.0
0.8559	0.958956217		0.009640153	0.0213	0.2075	0.3
0.7942	0.799666491		0.593556	0.0933	0.129	0.74
1.3706	1.954915503		0.000109636	0.0428	0.4765	0.7
1.3448	2.080200064		5.45E-05	0.0275	0.3679	0.72
1.3968	1.981184102		9.75E-05	0.0473	0.5445	0.75
0.8713	0.869041035		0.0356042	0.0008	######	0.0
1.0839	0.810208087		0.00046706	0.1647	0.3473	0.0
1.0273	0.778329292		0.05053176	0.0026	0.0019	0.04
0.9984	1.004418288		0.9583704	0.6608	0.7144	0.80
0.9642	1.007757879		0.9601651	0.9715	0.7049	0.18
1.0966	1.372242917		0.002968533	0.0022	0.0005	0.43
1.4232	2.600579211		0.3353777	0.0238	######	0.0
2.7363	3.005440446		0.000107827	0.005	0.0055	0.5
1.7044	1.303454577		0.005023932	0.035	0.0002	0.0
0.9747	1.016993365		0.2032325	0.9619	0.709	0.40
1.0203	0.999083548		0.1980279	0.5769	0.5012	0.4
1.6647	1.607715034		0.04581127	0.0134	0.0408	0.04
1.0573	1.076187249		0.01281673	0.0261	0.0709	0.02
1.7831	2.646153935		0.001077468	0.0247	0.2883	0.02
1.0001	1.065598949		0.3898523	0.8872	0.517	0.94
1.0074	0.967714224		0.000724491	0.0011	######	0.0
1.499	1.332019463		0.000328694	0.0004	0.0001	0.0
0.9882	0.956473743		0.0350447	0.0079	0.2318	0.0
1.1108	1.285074919		0.001864797	0.1236	0.018	0.65
0.8301	0.723330287		0.1845474	0.0738	0.1021	0.1
1.1348	1.560160635		0.00023843	0.0065	0.1164	0.0
1.0144	1.069752243		0.07161631	0.2012	0.003	####
1.0053	1.041075432		0.2682464	0.0569	0.0414	0.0
0.7722	1.125083787		3.94E-05	0.0124	0.0031	0.0
1.1125	1.527598974		0.004200588	0.8946	0.0009	0.96
1.0372	1.077795636		0.1264069	0.894	0.7846	0.
1.0396	0.988491975		0.7484926	0.0474	0.1452	0.9
0.8463	0.632816218		0.1655635	0.2333	0.0013	0.04
1.0006	0.974291749		0.02931733	0.1832	0.9856	0.06
1.0024	1.041352769		0.2318954	0.4966	0.757	0.
0.9591	0.987008953		0.4482822	0.0351	0.0001	0.0
1.0544	1.052328498		0.8354674	0.219	0.0022	0.04
0.7801	0.957762831		0.006157403	0.1769	0.7361	0.1
0.886	0.902119226		0.007771193	0.0043	0.0264	0.37
1.0465	1.066495947		0.7621901	0.5149	0.3043	0.5
1.0259	0.981078975		0.7820762	0.5412	0.3422	0.6
0.9735	1.004631261		0.9307217	0.6966	0.5588	0.76
1.0032	0.997781213		0.9396286	0.9193	0.8101	0.94
0.9568	0.807073696		0.004465434	0.4465	0.0272	0.63
0.8033	0.797062081		0.03896334	0.0009	0.1311	0.2
1.0295	0.995219467		0.000172662	0.5363	0.6507	0.74
1.0045	0.988242847		0.879897	0.264	0.3335	0.27
1.0833	1.188352646		0.657131	0.0166	0.0023	0.0

4. Insert dataset descriptors - In the top cell insert a name for the population followed by FC, in the second the country of the resistant population, in the third either Exposed or Unexposed dependent upon whether the resistant population is exposed, in the fourth the species Anopheles gambiae, Anopheles coluzzi or Anopheles arabiensis (MUST be full species ID) and in the fifth cell down the class of insecticide or 'None' if unexposed. Underneath this paste the raw fold changes corresponding to the probe of this row. Repeat this with Q values in the furthest right column on the sheet, keeping all information in the top 5 columns identical whilst replacing FC with Q.
5. Mapping data - Now open geography.txt, it will contain a column of resistant population names, exactly how they appear in the Q value columns of Fold Changes.txt, a latitude and a longitude. Input your population name EXACTLY as it appears in the Q value column under the last row of the first column, followed by the latitude and longitude of the collection site (or approximate original location) of the new resistant population in the dataset.
6. Install – replace the existing files with your newly modified files (Fold Changes.txt, geography.txt) and restart the application.

Section 3: Adapting the App to Handle Other Expression Data

Part of the utility of this app is use in a wider field than insecticide resistance alone. It will specifically be useful in fields that have a variety of transcriptomic data from different experiments, from which there will be some merit to analyse them together. To achieve this, there will need to be changes to the key code. For the purposes of this walkthrough, no previous knowledge of R is necessary but to fully adapt the code, R knowledge will be required. Due to this, the walkthrough will cover inputting data with ONLY 4 filtering criteria.

Creating a new data file

The first task is to create a new Fold Changes.txt file (tab delimited) to appropriately match the template provided for insecticide resistance as seen below.

PROBE LIST	Population0FC	Population1FC	Population0Q	Population1Q
FILTER 1				
FILTER 2				
FILTER 3				
FILTER 4				
Probe 1				
Probe 2				
Probe 3				
etc				

Populating the data file

Enter the appropriate parameters for each filter, as in the example below. Capitalisation and punctuation is important in R so make sure everything is consistent and DO NOT change, for example, between 'female' and 'Female'. These filters should overlap otherwise you will not be able to select multiple datasets

PROBE LIST	Patient0FC	Patient1FC	Patient0Q	Patient1Q
FILTER 1	Female	Male	Female	Male
FILTER 2	Caucasian	African American	Caucasian	African American
FILTER 3	Infected	Uninfected	Infected	Uninfected
FILTER 4	Treated	Treated	Treated	Treated
Probe 1	0.5	0.7	0.002	0.043
Probe 2	1.23	2.45	0.062	0.1254
Probe 3	10.2	1.24	0.00004	0.245
etc	5.4	2.6	0.006	0.032

Modifying the R code to accept your data

Once the data is in with the probes matching across rows for all datasets, the R code can now be modified by following the steps below.

The guide will assume that geography is NOT relevant to these datasets, so the map will be removed, as well as geography.txt. If geography is relevant ignore step a.) and the final step deleting lines 479-584 and modify the geographical parameters as in section (ii).

a. Open the R code in a text editor, delete the line *library(dismo)* and the line *geography<-read.delim('geography.txt',header=T)*
b. *titlePanel('IR-TEx', windowTitle = 'IR-TEx')* Change the name within the ' to your own dataset ie *titlePanel('Patient Data', windowTitle = 'Patient Data')*
c. *textInput('textInput','Transcript ID',value='AGAP008212-RA')* Change the AGAP008212-RA to a probe on your array, ie in the above example *textInput('textInput','Transcript ID',value='Probe 1')*. It must be exact.
d. *checkboxGroupInput('CountryInput','Select Relevant Countries',c('Burkina Faso','Cote D`Ivoire','Cameroon','Equatorial Guinea','Zambia','Tanzania','Sudan','Uganda','Togo'),selected=c('Burkina Faso','Cote D`Ivoire','Cameroon','Equatorial Guinea','Zambia','Tanzania','Sudan','Uganda','Togo'))* Change this line to your appropriate filters and those you wish to be selected when the app opens. For the example above this would become *checkboxGroupInput('CountryInput','Select Relevant Sex',c('Male','Female'),selected=c('Male','Female'))* This must correspond to the first row of filters, don't change 'CountryInput'
e. checkboxGroupInput('ExposureInput','Select Exposure Status',c('Exposed','Unexposed'),selected=c('Exposed','Unexposed')) Change this to correspond to filter 2 checkboxGroupInput('ExposureInput','Select Ethnicity Status',c('Caucasian','African American'),selected=c('Caucasian','African American')) This must correspond to the second row of filters, don't change 'ExposureInput'
f. checkboxGroupInput('SpeciesInput','Select Relevant Species',c('Anopheles gambiae','Anopheles coluzzi','Anopheles arabiensis'),selected = c('Anopheles coluzzi')) Change this to correspond to filter 3 checkboxGroupInput('SpeciesInput','Select Infectious Status',c('Infected','Uninfected'),selected = c('Infected','Uninfected')) This must correspond to the third row of filters, don't change 'SpeciesInput'
g. checkboxGroupInput('InsecticideInput','Select Insecticide Class',c('Pyrethroid','Organochloride','Carbamate','None'),selected = c('Pyrethroid','None')) Change this to correspond to filter 4 checkboxGroupInput('InsecticideInput','Select Treatment Regime',c('Treated','Untreated'),selected = c('Treated','Untreated')))) This must correspond to the fourth row of filters, don't change 'InsecticideInput'

There can be as many filters as required, each must be surrounded by 'X' and separated by a comma. The above correspond to lines 1-47 of the code. We will now scroll to lines 479-584. These will be deleted and begin/end to:

```
START: output$Geography <- renderPlot({
END    paste("Significant Transcripts Only (p", as.expression("<="),"0.05): FC > 5 = Red, FC > 1 = Amber, FC < 1 = Green",sep="")
  })
```

Inclusive of the brackets: })

Use of one colour arrays and RNASeq data with the app

One-colour arrays have often been used due to the original high price of two-colour arrays. Similarly, different arrays and different analysis techniques are often used on array data. With the advent of RNAseq data, this leads to a further confounding variable when using this app. Each of these techniques have different distributions of fold change, below are examples of the fold change distributions of four different experiments from (a) two colour arrays, (b) one colour arrays and (c) RNAseq. In each case fold change is on the x axis and the y axis represents the number of transcripts for each fold change.

(a)

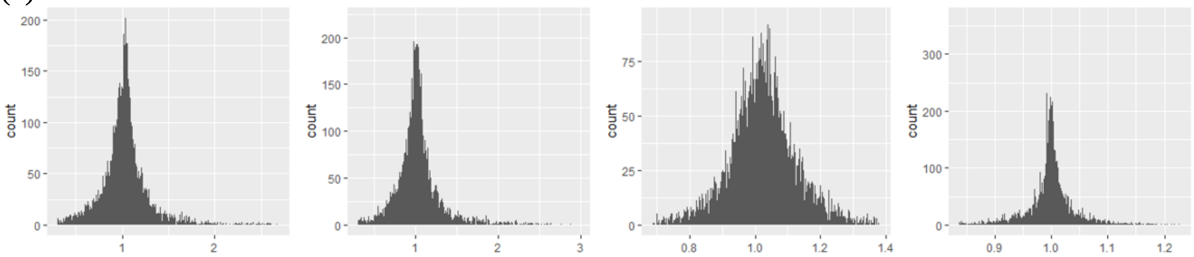

(b)

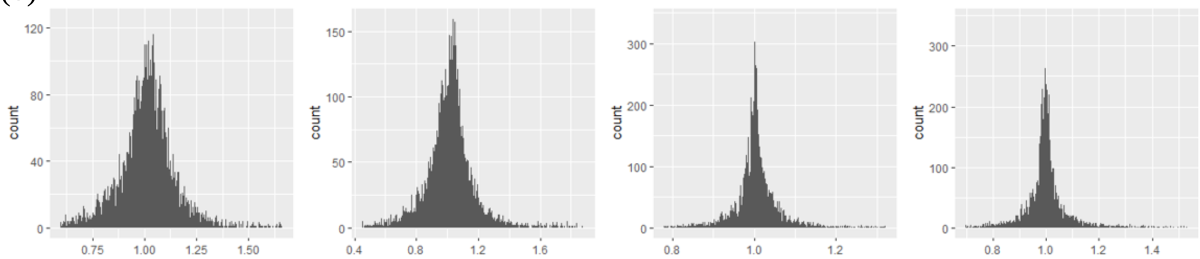

(c)

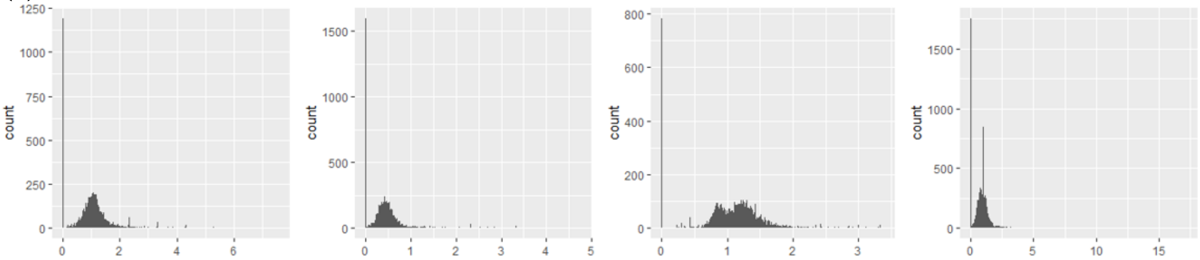

These differences in distributions are a confounding factor when integrating this data, specifically with use of correlation networks which are driven by extreme values, as often seen in RNAseq. To input data, follow guide in section ii, once this is complete and the data stored in a tab delimited text file, normalisation can be performed. By using a quantile normalisation across a table containing all fold changes, it is possible to make the distributions identical in terms of statistical properties and is a technique widely employed with microarray data analysis. As seen below, these distributions are much more similar after normalisation; this transformation can also be used across array platforms (Affymetrix, Agilent, Ilumina etc.)

(a)

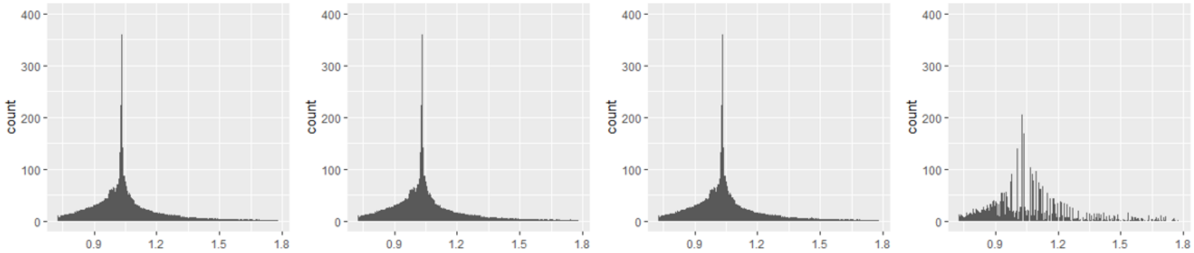

(b)

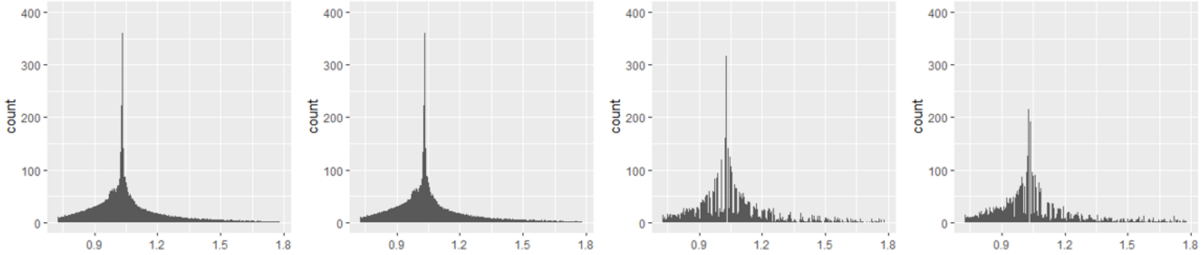

(c)

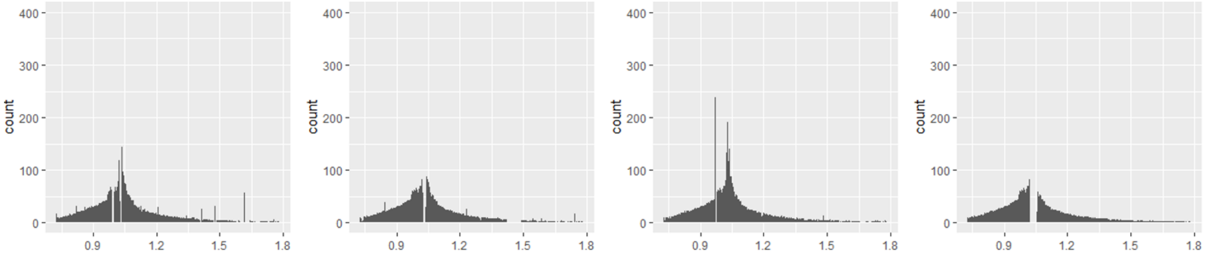

These data are kindly contributed by Duo Peng of the Catteruccia lab, Harvard T.H Chan School of Public Health.

Section 4: References

For further information about algorithms and a description of the methods and use case please see our publication "**Transcriptomic meta-signatures identified in *Anopheles gambiae* populations reveal previously undetected insecticide resistance mechanisms**" V.A Ingham, S. Wagstaff and H. Ranson. Nature Comms.

Where to find help

To install IR-TEx, you will need to download the current file from https://github.com/LSTMScientificComputing/IR-TEx and execute it in a ShinyR environment.

Detailed instructions how to deploy the ShinyR environment can be found on the Shiny project webpage - https://shiny.rstudio.com. Example instructions on how to configure ShinyR for Ubuntu can be found here.

Example tutorial on installing for Ubuntu 16.04
https://www.digitalocean.com/community/tutorials/how-to-set-up-shiny-server-on-ubuntu-16-04

Example tutorial on installing for Ubuntu 14.04
https://www.digitalocean.com/community/tutorials/how-to-set-up-shiny-server-on-ubuntu-14-04

Version

SW, VAI, HR authored the user guide
Data for different -omics platforms provided by DP
Created on: 20th August 2018
Updated on: 19th October 2018
Version: 1.1
